# Stochastic delay derivatives of Newcastle disease application in epidemic model: Stability analysis and approximation

**DOI:** 10.1371/journal.pone.0357918

**Published:** 2026-09-17

**Authors:** Naveed Shahid, Ali Raza, Marek Lampart, Sana Iqbal, Nauman Ahmed, Eman Ghareeb Rezk

**Affiliations:** 1 Department of Mathematics and Statistics, The University of Lahore, Lahore, Pakistan; 2 Department of Mathematics, Firat University, Elazığ, Türkiye; 3 IT4Innovations, VSB-Technical University of Ostrava, Ostrava, Czech Republic; 4 Research Center of Applied Mathematics, Khazar University, Baku, Azerbaijan; 5 Mathematical Science Department, College of Science, Princess Nourah bint Abdulrahman University, Riyadh, Saudi Arabia; Universidad Nacional de Colombia, COLOMBIA

## Abstract

The illicit trade in wildlife for pet purposes poses a direct risk to animal populations through overharvesting, but it also serves as an indirect conduit for the spread of contagious diseases. The present study assessed the effects of the hypothetical release of captured, infected individuals of Newcastle disease into the natural population of the white-winged parakeet, the most trafficked psittacine species in Peru. This study analysed the computational dynamical analysis of the stochastic susceptible-exposed-infected-recovered model of Newcastle disease. We take two approaches to stochastic modelling: transition probabilities and the perturbation method. To investigate dynamical features such as positivity, boundedness, consistency, and stability, we introduced a stochastic non-standard finite-difference (SNSFD) scheme. Conventional numerical approaches such as Euler-Maruyama, stochastic Euler, and stochastic Runge–Kutta of order four were applied, but they failed to preserve the system’s essential dynamical properties. Consequently, we formulated the SNSFD method to address this limitation. To support the proposed method, we presented several theorems that demonstrate it satisfies all dynamical properties of the model. In the end, we presented several simulations to compare the proposed method’s efficiency to that of an existing method.

## 1. Introduction

The exporting nation’s disease status and the types of products are two of the many variables that affect the risk of transmitting modifiable avian influenza (NAI) through trade. The trade in live birds is the most dangerous. To make sure that international trade does not lead to the spread of NAI, it is critical to evaluate and manage these risks [1]. Numerous species of free-ranging wild birds have experienced observable mortality due to outbreaks of clinical illness in Eurasia. The way influenza viruses circulate in natural environments is driven by selection pressure that favours low-pathogenicity variants, which are indirectly spread via waterbird droppings and contaminated fomites [2]. Australia should not assume that the epidemiology of AIV in other geographic regions applies here, given the country’s isolation, both geographic and environmental. The widespread discovery of H5 subtypes in wild birds may pose an ongoing risk to the Australian poultry sector, even though H7 subtypes have been linked to all prior highly virulent avian influenza outbreaks in Australian poultry [3]. Subtypes H6 and H9 of the avian influenza virus were frequently detected in the study population. The idea that there is a biosecurity danger is supported by the likelihood that one of the H6 viruses was recently introduced by migratory birds. Another H6 virus’s matrix gene resembled the H7 subtype found in Australia, indicating the assortment of a previously introduced H6 virus with native viruses [4]. In [5], Joy et al. investigated the optimal control of Newcastle disease and found that, without control strategies, the number of infected birds increased, whereas implementing control measures significantly reduced the proportion of infections. This work is a quantitative investigation into the role of cells in the activation of two measurable properties of Newcastle disease virus: enzymatic activity and infectivity by antibodies [6]. The main domestic and wild bird species are affected by the infectious disease known as Newcastle disease. Ordinary differential equation theory is used to develop and analyze a deterministic compartmental model for ND. Therefore, Markov Chain Monte Carlo (MCMC) simulations were used to investigate the uncertainties in model parameters using data on Newcastle disease-related chicken deaths from five districts in two regions of Tanzania [7]. Based on the findings of this study, the estimated ND incidence in eastern Zambia is on the rise each year, and this trend is expected to persist for the next 25 years, it further indicates that the primary economic policies in place from 1989 to 2014, which were used to undertake cattle disease control programs in this region, most likely had little effect on ND trends [8]. This study reviews the epidemiology of Newcastle disease in Nigerian village poultry, focusing on the age, species and type susceptibilities. The opportunities and challenges of controlling Newcastle disease in this Nigerian village chicken industry are also examined. This study aimed to develop efficient approaches for managing the illness in Nigeria’s rural communities [9]. The current study found that samples from the four markets in Kubwa and Lugbe had a high prevalence of haemagglutination antibodies. This suggests that the area is endemic for Newcastle disease virus infection and that the markets act as hubs for the spread of susceptible and infected birds. The buyers, sellers and meat processors are all potential conduits for the disease to spread [10]. In rural poultry in Kumasi and its surroundings, this study demonstrates subclinical Newcastle disease activity that may quickly progress to clinical disease under stress. Birds from houses may have multiple ND virus infections, which could explain their higher ND titers compared with samples obtained from the live-bird market [11]. In both commercial and village-rearing methods, Newcastle disease is a significant barrier to chicken production in Africa. A review of case records of Newcastle and other poultry diseases diagnosed at veterinary clinics in Kaduna, Nigeria, was conducted over 10 years [12]. In the Agarfa and Sinana districts of the Bale Zone, hens collected from backyards and small-scale poultry producer farms circulated antibodies of ND, according to the current serological investigation [13]. This study in Kindo Koisha district, Wolaita Zone, southern Ethiopia, revealed the incidence of circulating NDV in backyard poultry. Backyard poultry production systems are used to manage 89% of Ethiopia’s total population. Farmers typically raise native chicken breeds in their backyard [14]. This study illustrated the ND condition in backyard hens, emphasizing the need for a thorough immunization schedule for backyard hens [15]. In this study, backyard, semi-intensive and intensive farms in central and southwest Ethiopia will have their seropositivity levels and Newcastle disease virus levels measured [16]. Our findings indicate that Newcastle disease is prevalent among family flocks across the study area, with similar impact observed in both woredas and kebeles, though cases are more pronounced within villages. Seropositivity to NDV and exposure risk have been significantly associated with flock replenishment practices, flock size, cleaning frequency, water source, and the provision of grain supplements [17–20]. Recent studies have employed fractional and advanced numerical techniques for analyzing infectious-disease models. Khader and Adel investigated a fractional lumpy-skin-disease model using fractional Bernoulli functions, while Adel et al. examined a fractional coronavirus model. Finite-element and multidomain spectral relaxation methods have also been applied successfully to COVID-19 models. These contributions emphasize the importance of reliable computational approaches in epidemic modeling and support the development of the stochastic numerical method proposed in this study [21–24].

Shifting our focus from a potential mathematical framework that might explain the transmission dynamics of infections such as Newcastle disease, we turn to the current study. Since multiple populations are present and their interactions are complex, the mathematical model is deterministic. We will analyze the general growth of this system depicted as a stochastic differential equation, thus introducing randomness. This work attempts to understand the dynamics of infectious diseases, in particular the Newcastle virus, through stochastic modeling. It organizes the analysis around the presentation of the Stochastic Delay Model, which will be treated in the next sections. This makes a certain shift from a purely deterministic consideration of an infectious disease and its influence on physical processes toward a more probabilistic and realistic one. The given model is the SEIR system, divided into several smaller groups: S for susceptible, E for exposed, 𝐼𝐴 for acutely infected, 𝐼𝐶 for chronically infected, and 𝑅 for recovered. The structure of this paper is organized as follows: Section 2 discusses the dynamical properties of the model. Section 3 focuses on transition probabilities, parametric perturbations, and reproduction numbers. Section 4 presents the numerical investigations, including the SNSFD scheme, convergence analysis, and a comparative study of the stochastic model. Finally, Section 5 provides the concluding remarks.

## 2. Formulation of the delayed mathematical model

Newcastle disease virus can be transmitted through direct contact with the respiratory secretions and faecal material of infected birds and indirectly through contaminated food, water, equipment, and environmental materials. Infected birds may shed the virus during the incubation period and for a certain period during recovery, while prolonged viral shedding has been documented in some wild and psittacine birds. These epidemiological characteristics motivate the inclusion of both acute and prolonged infectious stages in the present model [25, 26]. The total white-winged parakeet population at time t is divided into five mutually exclusive epidemiological compartments,


$$\begin{array}{c} N(t)=S(t)+E(t)+I_{A}(t)+I_{C}(t)+R(t). \end{array}$$

Here, S(t) denotes susceptible parakeets that are not infected and do not possess sufficient effective immunity against Newcastle disease. The class E(t) consists of birds that have acquired the infection but have not yet progressed to an infectious state. Thus, $\frac{\text{1}}{\alpha }$ represents the average duration of the latent or pre-infectious stage. The inclusion of this class is epidemiologically reasonable because Newcastle disease has a nonzero incubation period, the duration of which depends on the viral strain, host species, immune status, and route of exposure. The class $I_{A}(t)$ represents acutely infected parakeets. These birds are assumed to exhibit a comparatively severe and highly infectious form of Newcastle disease and to shed relatively large amounts of virus through respiratory and faecal routes. The class $I_{C}(t)$ represents chronically or persistently infected birds that continue to shed the virus for a longer period but generally at a lower level than acutely infected individuals. Therefore, both $I_{A}(t)$ and $I_{C}(t)$ contribute to the force of infection, although it is biologically expected that $\beta_{A}>\beta_{C},$ where $\beta_{A}$ and $\beta_{C}$ are the transmission coefficients associated with acute and chronic infection, respectively. The $I_{C}$ compartment should not be interpreted as indicating a permanently diseased bird. Instead, it represents a prolonged infectious or lower-intensity shedding state. Experimental studies involving psittacine birds have demonstrated considerable variation in clinical outcomes and have reported prolonged excretion of Newcastle disease virus in parrots and related species [27]. Exposed birds progress to an infectious state at the rate α. A proportion $p_{\text{1}}$ develops acute infection and enters $I_{A}$, whereas the remaining proportion $\text{1}-p_{\text{1}}$ enters the chronic infectious class $I_{C}$. The use of these two pathways accounts for heterogeneity in host susceptibility, immune response, clinical severity, and viral shedding. Acutely infected birds leave $I_{A}$ at the rate δ. Among them, the proportion $p_{\text{2}}$ successfully clears the infectious state and enters R, while the proportion $\text{1}-p_{\text{2}}$ develops prolonged infection and enters $I_{C}$. Chronically infected birds recover at the rate γ. This branching structure is consistent with the previously proposed Newcastle disease transmission framework for white-winged parakeets, in which newly infected birds could develop either acute or chronic infection and some acutely infected birds subsequently entered a prolonged infectious state. The recovered compartment R(t) contains birds that have cleared active infection and possess effective post-infection protection. The transition $R\overset{\nu }{\to }S$ represents the gradual loss of effective protection rather than the complete removal of all immunological memory. Thus, $\frac{\text{1}}{\nu }$ is interpreted as the average duration for which recovered birds remain sufficiently protected against reinfection. A finite duration of effective immunity has previously been incorporated into a Newcastle disease model for white-winged parakeets based on experimentally measured effective antibody titres [21]. Nevertheless, because longitudinal immunity data for free-ranging white-winged parakeets remain limited, ν is treated as an uncertain parameter. The special case ν=0 corresponds to permanent immunity and may be considered in sensitivity analyses. The term $\left[\beta_{A}S(t-\tau )I_{A}(t-\tau )+\beta_{C}S(t-\tau )I_{C}(t-\tau )\right]e^{-(d+h)\tau }$describes delayed transmission generated by acute and chronic infectious birds. The delay τ is interpreted as an effective transmission-establishment delay, i.e., the interval between infectious contact and the establishment of a newly infected individual. It is distinguished from the subsequent progression of an exposed bird at the rate α. The factor $e^{-\;(d+h)\tau }$ represents the probability that an individual survives natural mortality and harvest-related removal during the delay period.

**Fig 1:**
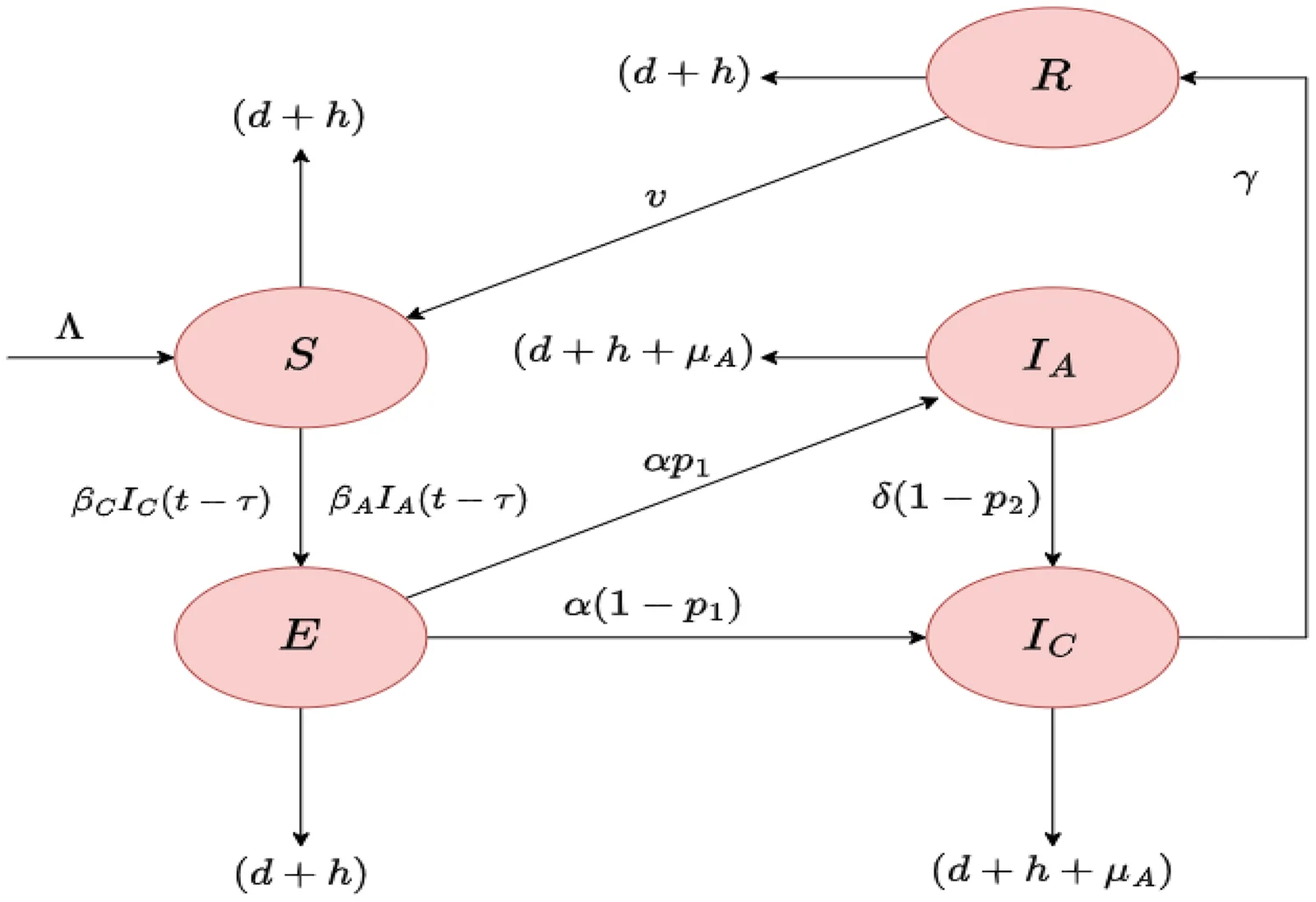
Flow dynamics of New-Castle disease.

The model is constructed under the following simplifying assumptions: the parakeet population mixes homogeneously; all susceptible birds have the same probability of effective contact; the transmission process is density dependent; both acute and chronic infectious birds can transmit the virus; recruitment occurs directly into the susceptible class; natural mortality and harvest affect all compartments; disease-induced mortality occurs only in the infectious classes; and vaccination, vertical transmission, immigration, emigration, age structure, and seasonal changes are not explicitly included. These assumptions provide a mathematically tractable framework for studying stochastic and delayed dynamics of Newcastle disease Fig 1. However, they also represent limitations to consider when interpreting the numerical results.

The parameter values, units, sources, and biologically admissible ranges are summarized in Table 1. Newcastle disease parameters were taken or adapted from published studies on white-winged parakeets and related avian populations. The current definition of α as the “rate at which infection spread” should particularly be corrected because transmission is governed by $\beta_{A}$ and $\beta_{C}$, whereas α governs progression from exposure to infectiousness

**Table 1:** Description of model parameters.

Λ	Represents the birth rate of the population
α	Progression rate from the exposed class to the infectious classes; 1/α is the mean latent period
δ	Rate of leaving the acute infectious class
γ	Recovery rate from the chronic infectious class
$\beta_{A}$	Transmission coefficient associated with acutely infected birds
$\beta_{C}$	Transmission coefficient associated with chronically infected birds, generally with $\beta_{C}<\beta_{A}$
$\mu_{A}$	Newcastle-disease-induced mortality rate in acutely infected birds
$\mu_{C}$	Newcastle-disease-induced mortality rate in chronically infected birds
$p_{\text{1}}$	Probability that an exposed bird develops acute infection
$\text{1}-p_{\text{1}}$	Probability that an exposed bird develops chronic infection directly
$p_{\text{2}}$	Conditional probability that an acutely infected bird recovers
$\text{1}-p_{\text{2}}$	Conditional probability that an acutely infected bird progresses to chronic infection
ν	Rate of loss of effective post-infection protection
τ	Effective transmission-establishment delay
d	Natural mortality rate
h	Harvest or human-related removal rate

To derive the stochastic delay model τ for the disease spread in the parakeet population, many assumptions about the behavior of each community will be necessary. On the other hand parameters of the model are described as in Table 1 and are non-negative. The given model differential equation is shown below:


$$\begin{array}{c} \frac{dS}{dt}=\Lambda -(d+h)S(t)-\left(\beta_{A}S(t-\tau )I_{A}(t-\tau )+\beta_{C}S(t-\tau )I_{C}(t-\tau )\right)e^{-(d+h)\tau }+\nu R(t), \end{array}$$ (1)

$$\begin{array}{c} \frac{dE}{dt}=\left(\beta_{A}S(t-\tau )I_{A}(t-\tau )+\beta_{C}S(t-\tau )I_{C}(t-\tau )\right)e^{-(d+h)\tau }-(d+h+\alpha )E(t), \end{array}$$ (2)

$$\begin{array}{c} \frac{dI_{A}}{dt}=\alpha p_{\text{1}}E(t)-(d+h+\mu_{A}+\delta )I_{A}(t), \end{array}$$ (3)

$$\begin{array}{c} \frac{dI_{C}}{dt}\;=\;\alpha \left(\text{1}-p_{\text{1}}\right)E(t)+\delta \left(\text{1}-p_{\text{2}}\right)I_{A}(t)-\left(d+h+\mu_{C}+\gamma \right)I_{C}(t), \end{array}$$ (4)

$$\begin{array}{c} \frac{dR}{dt}=\;\delta p_{\text{2}}I_{A}+\gamma I_{C}-(d+h+\nu )R(t),\; \end{array}$$ (5)

$N(t)=S(t)+E(t)+I_{A}(t)+I_{C}(t)+R(t),\forall \;t\geq \text{0}.$ The prerequisites are as follows:


$$\begin{array}{c} \;S(\text{0})\geq \text{0},\;E(\text{0})\geq \text{0},\;I_{A}(\text{0})\geq \text{0},\;I_{C}(\text{0})\geq \text{0},\;R(\text{0})\geq \text{0}\text{.} \end{array}$$ (6)

### 2.1. Feasible properties of the model

Let $X(t)=\;{\left(S(t),\;E(t),\;I_{A}(t),\;I_{C}(t),\;R(t)\right)}^{T}$ and assume that the initial history belongs to $C([-\tau ,\;\text{0}],\;R_{+}^{5})$. Since the right-hand side of the delayed system is continuous in time and locally Lipschitz continuous with respect to the state variables on every bounded subset of the non-negative state space, the standard existence and uniqueness theorem for delay differential equations guarantees a unique local solution corresponding to each admissible initial history.

To establish positivity, observe that the model is quasi-positive. Specifically, whenever one state variable is zero and all remaining state variables are non-negative, the corresponding derivative is non-negative. Therefore, the vector field points inward on each boundary face of the non-negative orthant. It follows that any solution starting from non-negative initial data remains non-negative for all times for which the solution exists.

DefSame as for function by


$$\begin{array}{c} N(t)=S(t)+E(t)+I_{A}(t)+I_{C}(t)+R(t). \end{array}$$

Adding the five model equations and cancelling the internal transfer terms yields


$$\begin{array}{c} \frac{dN(t)}{dt}\;=\;\Lambda \;-\;(d\;+\;h)N(t)-\;\mu_{A}\;I_{A}(t)-\;\mu_{C}\;I_{C}(t). \end{array}$$

Because $I_{A}(t)\;\geq \;\text{0}$ and $I_{C}(t)\;\geq \;\text{0}$, we obtain the differential inequality


$$\begin{array}{c} \frac{dN(t)}{dt}\;\leq \;\Lambda \;-\;(d\;+\;h)N(t). \end{array}$$

The comparison theorem therefore gives


$$\begin{array}{c} N(t)\leq \;N(\text{0})e^{\left[-(d+h)t\right]+\;\left[\frac{\Lambda }{d+h}\right]\left(\text{1}\;-\;e^{\left[-(d+h)t\right]}\right)} \end{array}$$ (7)

Consequently,


$$\begin{array}{c} N(t)\;\leq \;max\{N(\text{0}),\;\Lambda /(d+h)\}. \end{array}$$

Hence, if $N(\text{0})\leq \frac{\Lambda }{d+h}$, then $N(t)\leq \frac{\Lambda }{d+h}$ for every t≥0. Thus, the biologically feasible region


$$\begin{array}{c} \Omega \;=\;\left\{X\;\in R^{\text{5}+}:\;S+E+I_{A}\;+\;I_{C}\;+R\leq \frac{\Lambda }{d+h}\right\} \end{array}$$

is positively invariant. Since all state variables remain non-negative and bounded in this region, finite-time blow-up cannot occur. Therefore, the local solution extends to a unique global solution defined for all t ≥ 0. This proves existence, uniqueness, non-negativity, boundedness, and positive invariance of the biologically feasible region.

***Lemma 1***: System (1)-(5) guarantees the existence and uniqueness of its solution for all t≥0.

***Proof***: The analysis proceeds by considering the Banach space with the norm defined by ${\left\|\lambda \right\|}_{\infty }={sup}_{t\epsilon D_{\lambda }}\left|\lambda (t)\right|$. To ascertain the properties of the solution to this problem, one must examine whether the system satisfies appropriate growth and Lipschitz conditions.

Let us consider three positive constants $M_{\text{1}},\;M_{\text{2}},M_{\text{3}},M_{\text{4}}\;$and $M_{\text{5}}<\infty$ such that $S_{\infty }<M_{\text{1}}$,$\;E_{\infty }<M_{\text{2}}$ ${I_{A}}_{\infty }<M_{\text{3}}$, ${I_{C}}_{\infty }<M_{\text{4}}$ and $R_{\infty }<M_{\text{5}}$.

We have,


$$\begin{array}{c} \left\{ \begin{array}{c} @lS^{\prime }=f_{\text{1}}(S,E,I_{A},I_{C},R,t)\\ E^{\prime }=f_{\text{2}}\left(S,E,I_{A},I_{C},R,t\right)\;\\ \begin{array}{c} {I_{A}}^{\prime }=f_{\text{3}}\left(S,E,I_{A},I_{C},R,t\right)\\ \begin{array}{c} {I_{C}}^{\prime }=f_{\text{4}}\left(S,E,I_{A},I_{C},R,t\right)\\ R^{\prime }=f_{\text{5}}(S,E,I_{A},I_{C},R,t) \end{array} \end{array} \end{array}\right.\;\;\;\;,\;\forall t\geq \text{0}. \end{array}$$ (8)

We verify that,


$$\begin{array}{c} {\left|f_{i}(S,t)\right|}^{\text{2}}<k_{i}\left({\left|S_{i}\right|}^{\text{2}}+\text{1}\right),\text{ Where }i=\text{1},\text{2},\text{3},\text{4},\text{5}. \end{array}$$ (9)

$$\begin{array}{c} {\left|f_{i}\left(S^{\text{1}},t\right)-f_{i}\left(S^{\text{2}},t\right)\right|}^{\text{2}}<\overset{―}{k_{i}}{\left|S^{\text{1}}-S^{\text{2}}\right|}^{\text{2}}. \end{array}$$ (10)

We examine the function $f_{\text{1}}\left(S,E,I_{A},I_{C},R,t\right)$ as evidence, along with the following assumptions that are upheld.


$$\begin{array}{c} {\left|f_{\text{1}}(S,E,I_{A},I_{C},R,t)\right|}^{\text{2}}=\;{\left|\Lambda -(d+h)S(t)-\left(\beta_{A}SI_{A}+\beta_{C}SI_{C}\right)e^{-(d+h)\tau }+\nu R(t)\right|}^{\text{2}}, \end{array}$$ (11)

$$\begin{array}{c} \;\leq |\Lambda {|}^{\text{2}}+{\left|(d+h)S(t)\right|}^{\text{2}}+{\left|\left(\beta_{A}SI_{A}+\beta_{C}SI_{C}\right)e^{-(d+h)\tau }\right|}^{\text{2}}+{\left|\nu R(t)\right|}^{\text{2}}, \end{array}$$

$$\begin{array}{c} \;\leq \left(|\Lambda {|}^{\text{2}}+{sup}_{t\epsilon D_{\lambda }}{\left|(d+h)S\right|}^{\text{2}}+{sup}_{t\epsilon D_{\lambda }}{\left|\left(\beta_{A}SI_{A}+\beta_{C}SI_{C}\right)e^{-(d+h)\tau }\right|}^{\text{2}},\;+{sup}_{t\epsilon D_{\lambda }}{\left|\nu R(t)\right|}^{\text{2}}\right). \end{array}$$

$$\begin{array}{c} \;\leq \left(|\Lambda {|}^{\text{2}}+|d+h{|}^{\text{2}}{\left\|S\right\|}_{\infty }+{\left|\beta_{A}\right|\left\|S\right\|}_{\infty }{\left\|I_{A}\right\|}_{\infty }e^{-(d+h)\tau }+\left|\beta_{C}\right|{\left\|S\right\|}_{\infty }{\left\|I_{C}\right\|}_{\infty }e^{-(d+h)\tau }\;+|\nu {|}^{\text{2}}{\left\|R\right\|}_{\infty }\right), \end{array}$$

$$\begin{array}{c} {\left|f_{\text{1}}\left(S,E,I_{A},I_{C},R,t\right)\right|}^{\text{2}}\;\leq \left(|\Lambda {|}^{\text{2}}+|d+h{|}^{\text{2}}{\left\|S\right\|}_{\infty }+|\nu {|}^{\text{2}}{\left\|R\right\|}_{\infty }\right) \end{array}$$

$$\begin{array}{c} \left(\text{1}+\frac{{\left|\beta_{A}\right|\left\|S\right\|}_{\infty }{\left\|I_{A}\right\|}_{\infty }e^{-(d+h)\tau }+\left|\beta_{C}\right|{\left\|S\right\|}_{\infty }{\left\|I_{C}\right\|}_{\infty }e^{-(d+h)\tau }}{|\Lambda {|}^{\text{2}}+|d+h{|}^{\text{2}}{\left\|S\right\|}_{\infty }+|\nu {|}^{\text{2}}{\left\|R\right\|}_{\infty }}\right), \end{array}$$

where, $\left(\frac{{\left|\beta_{A}\right|\left\|S\right\|}_{\infty }{\left\|I_{A}\right\|}_{\infty }e^{-(d+h)\tau }+\left|\beta_{C}\right|{\left\|S\right\|}_{\infty }{\left\|I_{C}\right\|}_{\infty }e^{-(d+h)\tau }}{|\Lambda {|}^{\text{2}}+|d+h{|}^{\text{2}}{\left\|S\right\|}_{\infty }+|\nu {|}^{\text{2}}{\left\|R\right\|}_{\infty }}\right)<\text{1}$.


$$\begin{array}{c} {\text{So},\left|f_{\text{1}}(S,E,I_{A},I_{C},R,t)\right|}^{\text{2}}\;<k_{\text{1}}\left(\text{1}+|S{|}^{\text{2}}\right). \end{array}$$ (12)

Same as for function$f_{\text{2}}\left(S,E,I_{A},I_{C},R,t\right),\;then\;we\;get$


$$\begin{array}{c} {\left|f_{\text{2}}\left(S,E,I_{A},I_{C},R,t\right)\right|}^{\text{2}}=\;{\left|\left(\beta_{A}SI_{A}+\beta_{C}SI_{C}\right)e^{-(d+h)\tau }+(d+h+\alpha )E(t)\right|}^{\text{2}}, \end{array}$$

$$\begin{array}{c} {\left|f_{\text{2}}\left(S,E,I_{A},I_{C},R,t\right)\right|}^{\text{2}}\leq {\left|\beta_{A}SI_{A}e^{-(d+h)\tau }\right|}^{\text{2}}+{\left|\beta_{C}SI_{C}e^{-(d+h)\tau }\right|}^{\text{2}}+{\left|(d+h+\alpha )E\right|}^{\text{2}}, \end{array}$$

$$\begin{array}{c} {\left|f_{\text{2}}\left(S,E,I_{A},I_{C},R,t\right)\right|}^{\text{2}}\leq \end{array}$$

$$\begin{array}{c} \left({sup}_{\tau \epsilon D_{\lambda }}{\left|\beta_{A}SI_{A}e^{-(d+h)\tau }\right|}^{\text{2}}+{sup}_{\tau \epsilon D_{\lambda }}{\left|\beta_{C}SI_{C}e^{-(d+h)\tau }\right|}^{\text{2}}+{sup}_{\tau \epsilon D_{\lambda }}{\left|(d+h+\alpha )E\right|}^{\text{2}}\right), \end{array}$$

$$\begin{array}{c} {\left|f_{\text{2}}\left(S,E,I_{A},I_{C},R,t\right)\right|}^{\text{2}}\leq \end{array}$$

$$\begin{array}{c} \left|\beta_{A}\right|{\left\|S\right\|}_{\infty }{\left\|I_{A}\right\|}_{\infty }e^{-(d+h)\tau }+\left|\beta_{C}\right|{\left\|S\right\|}_{\infty }{\left\|I_{C}\right\|}_{\infty }e^{-(d+h)\tau }+{\left|(d+h+\alpha )\right|}^{\text{2}}{\left\|E\right\|}_{\infty }, \end{array}$$

$$\begin{array}{c} {\left|f_{\text{2}}\left(S,E,I_{A},I_{C},R,t\right)\right|}^{\text{2}}\leq \;{\left|(d+h+\alpha )\right|}^{\text{2}}E_{\infty }\left(\text{1}+\frac{\left|\beta_{A}\right|{\left\|S\right\|}_{\infty }{\left\|I_{A}\right\|}_{\infty }e^{-(d+h)\tau }+\left|\beta_{C}\right|{\left\|S\right\|}_{\infty }{\left\|I_{C}\right\|}_{\infty }e^{-(d+h)\tau }}{{\left|(d+h+\alpha )\right|}^{\text{2}}{\left\|E\right\|}_{\infty }}\right), \end{array}$$

where,


$$\begin{array}{c} \frac{\left|\beta_{A}\right|{\left\|S\right\|}_{\infty }{\left\|I_{A}\right\|}_{\infty }e^{-(d+h)\tau }+\left|\beta_{C}\right|{\left\|S\right\|}_{\infty }{\left\|I_{C}\right\|}_{\infty }e^{-(d+h)\tau }}{{\left|(d+h+\alpha )\right|}^{\text{2}}{\left\|E\right\|}_{\infty }}<\text{1}, \end{array}$$

$$\begin{array}{c} {\left|f_{\text{2}}\left(S,E,I_{A},I_{C},R,t\right)\right|}^{\text{2}}<k_{\text{2}}\left(\text{1}+|E{|}^{\text{2}}\right). \end{array}$$

Similarly for $f_{\text{3}}\left(S,E,I_{A},I_{C},R,t\right),f_{\text{4}}\left(S,E,I_{A},I_{C},R,t\right),f_{\text{5}}\left(S,E,I_{A},I_{C},R,t\right)$, we get


$$\begin{array}{c} {\left|f_{\text{3}}\left(S,E,I_{A},I_{C},R,t\right)\right|}^{\text{2}}\leq {\left|\left(d+h+\mu_{A}+\delta \right)\right|}^{\text{2}}{\left\|I_{A}\right\|}_{\infty }\left(\text{1}+\frac{\;{\left|\left(\alpha p_{\text{1}}\right)\right|}^{\text{2}}{\left\|E\right\|}_{\infty }}{{\left|\left(d+h+\mu_{A}+\delta \right)\right|}^{\text{2}}{\left\|I_{A}\right\|}_{\infty }}\right), \end{array}$$

where,


$$\begin{array}{c} \frac{\;{\left|\left(\alpha p_{\text{1}}\right)\right|}^{\text{2}}{\left\|E\right\|}_{\infty }}{{\left|\left(d+h+\mu_{A}+\delta \right)\right|}^{\text{2}}{\left\|I_{A}\right\|}_{\infty }}<\text{1}. \end{array}$$

$$\begin{array}{c} {\left|f_{\text{3}}\left(S,E,I_{A},I_{C},R,t\right)\right|}^{\text{2}}<\;k_{\text{3}}\left(\text{1}+{\left|I_{A}\right|}^{\text{2}}\right), \end{array}$$

$$\begin{array}{c} {\left|f_{\text{4}}\left(S,E,I_{A},I_{C},R,t\right)\right|}^{\text{2}}\leq {\left|\left(d+h+\mu_{A}+\delta \right)\right|}^{\text{2}}{\left\|I_{C}\right\|}_{\infty } \end{array}$$

$$\begin{array}{c} \left(\text{1}+\frac{\;{\left|\alpha \left(\text{1}-p_{\text{1}}\right)\right|}^{\text{2}}{\left\|E\right\|}_{\infty }+{\left|\delta \left(\text{1}-p_{\text{2}}\right)\right|}^{\text{2}}{\left\|I_{A}\right\|}_{\infty }}{{\left|\left(d+h+\mu_{C}+\gamma \right)\right|}^{\text{2}}{\left\|I_{C}\right\|}_{\infty }}\right), \end{array}$$

where,


$$\begin{array}{c} \frac{\;{\left|\alpha \left(\text{1}-p_{\text{1}}\right)\right|}^{\text{2}}{\left\|E\right\|}_{\infty }+{\left|\delta \left(\text{1}-p_{\text{2}}\right)\right|}^{\text{2}}{\left\|I_{A}\right\|}_{\infty }}{{\left|\left(d+h+\mu_{C}+\gamma \right)\right|}^{\text{2}}{\left\|I_{C}\right\|}_{\infty }}<\text{1}, \end{array}$$

$$\begin{array}{c} {\left|f_{\text{4}}\left(S,E,I_{A},I_{C},R,t\right)\right|}^{\text{2}}<k_{\text{4}}\left(\text{1}+{\left|I_{C}\right|}^{\text{2}}\right), \end{array}$$

$$\begin{array}{c} {\left|f_{\text{5}}\left(S,E,I_{A},I_{C},R,t\right)\right|}^{\text{2}}\leq {\left|(d+h+\nu )\right|}^{\text{2}}{\left\|R\right\|}_{\infty }\;\left(\text{1}+\frac{{\left|\delta p_{\text{2}}\right|}^{\text{2}}{\left\|I_{A}\right\|}_{\infty }+|\gamma {|}^{\text{2}}{\left\|I_{C}\right\|}_{\infty }}{{\left|(d+h+\nu )\right|}^{\text{2}}{\left\|R\right\|}_{\infty }}\right), \end{array}$$

where,


$$\begin{array}{c} \frac{{\left|\delta p_{\text{2}}\right|}^{\text{2}}{\left\|I_{A}\right\|}_{\infty }+|\gamma {|}^{\text{2}}{\left\|I_{C}\right\|}_{\infty }}{{\left|(d+h+\nu )\right|}^{\text{2}}{\left\|R\right\|}_{\infty }}<\text{1}. \end{array}$$

Also,


$$\begin{array}{c} {\left|f_{\text{5}}\left(S,E,I_{A},I_{C},R,t\right)\right|}^{\text{2}}<\;k_{\text{5}}\left(\text{1}+|R{|}^{\text{2}}\right) \end{array}$$

$$\begin{array}{cc} & max\{\frac{{\left|\beta_{A}\right|\left\|S\right\|}_{\infty }{\left\|I_{A}\right\|}_{\infty }e^{-(d+h)\tau }+\left|\beta_{C}\right|{\left\|S\right\|}_{\infty }{\left\|I_{C}\right\|}_{\infty }e^{-(d+h)\tau }}{|\Lambda {|}^{\text{2}}+|d+h{|}^{\text{2}}{\left\|S\right\|}_{\infty }+|\nu {|}^{\text{2}}{\left\|R\right\|}_{\infty }},\;\frac{\left|\beta_{A}\right|{\left\|S\right\|}_{\infty }{\left\|I_{A}\right\|}_{\infty }e^{-(d+h)\tau }+\left|\beta_{C}\right|{\left\|S\right\|}_{\infty }{\left\|I_{C}\right\|}_{\infty }e^{-(d+h)\tau }}{{\left|(d+h+\alpha )\right|}^{\text{2}}{\left\|E\right\|}_{\infty }},\\ & \frac{\;{\left|\left(\alpha p_{\text{1}}\right)\right|}^{\text{2}}{\left\|E\right\|}_{\infty }}{{\left|\left(d+h+\mu_{A}+\delta \right)\right|}^{\text{2}}{\left\|I_{A}\right\|}_{\infty }},\frac{\;{\left|\alpha \left(\text{1}-p_{\text{1}}\right)\right|}^{\text{2}}{\left\|E\right\|}_{\infty }+{\left|\delta \left(\text{1}-p_{\text{2}}\right)\right|}^{\text{2}}{\left\|I_{A}\right\|}_{\infty }}{{\left|\left(d+h+\mu_{C}+\gamma \right)\right|}^{\text{2}}{\left\|I_{C}\right\|}_{\infty }},\frac{{\left|\delta p_{\text{2}}\right|}^{\text{2}}{\left\|I_{A}\right\|}_{\infty }+|\gamma {|}^{\text{2}}{\left\|I_{C}\right\|}_{\infty }}{{\left|(d+h+\nu )\right|}^{\text{2}}{\left\|R\right\|}_{\infty }}\} \end{array}$$

<1.

as desired.

***Lemma 2***: (Positivity) For given data in Eq. (6), $\left(S(\text{0}),E(\text{0}),I_{A}(\text{0}),I_{C}(\text{0})\;R(\text{0})\right)\in R_{+}^{\text{5}}$, then the solution $\left(S(t),E(t),I_{A}(t),I_{C}(t),R(t)\right)$ for the system (1)-(5) is positive for all time t>0.

***Proof***: Assuming that, $S(\text{0})\geq \text{0},E(\text{0})\geq \text{0},I_{A}(\text{0})\geq \text{0},I_{C}(\text{0})\geq \text{0},$ and R(0)≥0.

So, from system (1) – (5), we have


$$\begin{array}{c} S^{\prime }=\Lambda -(d+h)S(t)-\left(\beta_{A}SI_{A}+\beta_{C}SI_{C}\right)e^{-(d+h)\tau }+\nu R(t), \end{array}$$

$$\begin{array}{c} S^{\prime }\geq -\left[(d+h)-\left(\beta_{A}I_{A}+\beta_{C}I_{C}\right)e^{-(d+h)\tau }\right]S, \end{array}$$

This implies that


$$\begin{array}{c} nS=-\left[(d+h)-\left(\beta_{A}I_{A}+\beta_{C}I_{C}\right)e^{-(d+h)\tau }\right]t+c, \end{array}$$

$$\begin{array}{c} S=e^{c}\times e^{-\left[(d+h)-\left(\beta_{A}I_{A}+\beta_{C}I_{C}\right)e^{-(d+h)\tau }\right]t} \end{array}$$

$$\begin{array}{c} S(t)=S(\text{0})\times e^{-\left[(d+h)-\left(\beta_{A}I_{A}+\beta_{C}I_{C}\right)e^{-(d+h)\tau }\right]t}\geq \text{0}. \end{array}$$

Similarly, for $E(\text{0})\geq \text{0},I_{A}(\text{0})\geq \text{0},I_{C}(\text{0})\geq \text{0},R(\text{0})\geq \text{0},$


$$\begin{array}{c} E(t)\geq E(\text{0})\times e^{-[d+h+\alpha ]t}\geq \text{0}, \end{array}$$

$$\begin{array}{c} I_{A}(t)\geq \;I_{A}(\text{0})\times e^{-\left[d+h+\mu_{A}+\delta \right]t}\geq \text{0}, \end{array}$$

$$\begin{array}{c} I_{C}(t)\geq \;I_{C}(\text{0})\times e^{-\left[d+h+\mu_{C}+\gamma \right]t}\geq \text{0}, \end{array}$$

$$\begin{array}{c} R(t)\geq R(\text{0})\times e^{-[d+h+\nu ]t}\geq \text{0}\text{.} \end{array}$$

Hence, these conditions show that $S(\text{0})\geq \text{0},E(\text{0})\geq \text{0},I_{A}(\text{0})\geq \text{0},I_{C}(\text{0})\geq \text{0},R(\text{0})\geq \text{0}$ are positive, for any time t.

### 2.2. Equilibrium points

We assume that the state variables are fixed when determining the system’s equilibrium points. The following illustrates the state of equilibrium for the system (1)-(5):

(i) Newcastle free equilibrium $=C_{\text{1}}=\left(S^{\text{1}},E^{\text{1}}{,I_{A}}^{\text{1}}{,I_{C}}^{\text{1}},\;R^{\text{1}}\right)=\left(\frac{\Lambda }{d+h},\text{0},\text{0},\text{0},\text{0}\right).$
(ii) Newcastle existing equilibrium $=C_{\text{2}}=\left(S^{*},\;E^{*}{,I_{A}}^{*}{,I_{C}}^{*},\;R^{*}\right)$,

where


$$\begin{array}{c} S^{*}=\frac{\Lambda +\nu R^{*}}{(d+h)+{\beta_{A}{I_{A\;}}^{*}e^{-(d+h)\tau }+\beta_{C}{I_{C}}^{*}e}^{-(d+h)\tau }},\;E^{*}=\frac{\left({\beta_{A}S^{*}{I_{A\;}}^{*}e^{-(d+h)\tau }+\beta_{C}S{I_{C}}^{*}e}^{-(d+h)\tau }\right)}{d+h+\alpha }, \end{array}$$

$$\begin{array}{c} {I_{A}}^{*}=\frac{\alpha p_{\text{1}}E^{*}}{d+h+\mu_{A}+\delta },{I_{C}}^{*}=\frac{\alpha {(\text{1}-p}_{\text{1}})E^{*}+\delta (\text{1}-p_{\text{2}}){I_{A}}^{*}}{d+h+\mu_{C}+\gamma },R^{*}=\frac{\delta p_{\text{2}}{I_{A}}^{*}+\gamma {I_{C}}^{*}}{d+h+\nu }. \end{array}$$

### 2.3. Reproduction number

The basic reproduction number, denoted by R₀, is defined as the expected number of secondary infections generated by one typical infected bird introduced into an otherwise susceptible population. The next-generation matrix method is applied below to derive R₀ rigorously for the Newcastle disease model.

1. Infected compartments and disease-free equilibrium

The epidemiologically infected subsystem consists of the exposed, acutely infected, and chronically infected classes. Although exposed birds do not yet transmit infection, they are included because they contain infected individuals who subsequently progress to one of the infectious classes. Therefore, the infected-state vector is


$$\begin{array}{c} x={\left(E,I_{A},I_{C}\right)}^{T}. \end{array}$$

At the disease-free equilibrium, all infected compartments vanish and the susceptible population is determined by the balance between recruitment and removal.

Hence, $\varepsilon_{\text{0}}=\left(S_{\text{0}},\text{0},\text{0},\text{0},\text{0}\right),$ where $S_{\text{0}}=\frac{\Lambda }{d+h}$.

For compactness, define the total exit rates from the exposed, acute-infection, and chronic-infection compartments as follows:

$a_{E}=d+h+\alpha$, $a_{A}=d+h+\mu +\delta$, $a_{C}=d+h+\mu_{C}+\gamma$.

2. New-infection and transition terms

Following the next-generation approach, the infected subsystem is written as the difference between the appearance of new infections and all other transfers among infected compartments:


$$\begin{array}{c} \frac{dx}{dt}=F(x)-V(x), \end{array}$$

Only the exposed compartment receives newly infected birds. The acute and chronic classes receive individuals through progression from existing infected compartments; therefore, these transfers are not classified as new infections. The new-infection vector is


$$\begin{array}{c} F(x)=(\begin{array}{c} Se^{-(d+h)\tau }\left(\beta_{A}I_{A}+\beta_{C}I_{C}\right)\\ \begin{array}{c} \text{0}\\ \text{0} \end{array} \end{array}) \end{array}$$

The remaining transition vector, including progression, recovery, disease-induced mortality, natural mortality, and harvesting, is


$$\begin{array}{c} V(x)=(\begin{array}{c} a_{E}E\\ a_{A}I_{A}-\alpha p_{\text{1}}E\\ a_{C}I_{C}-\alpha \left(\text{1}-p_{\text{1}}\right)E-\delta \left(\text{1}-p_{\text{2}}\right)I_{A} \end{array}). \end{array}$$

3. Construction of the next-generation matrices

The Jacobian matrices of the new-infection and transition vectors are evaluated at the disease-free equilibrium. This gives


$$\begin{array}{c} F=DF\left(\varepsilon_{\text{0}}\right)=(\begin{array}{ccc} \text{0} & \beta_{A}S_{\text{0}}e^{-(d+h)\tau } & \beta_{C}S_{\text{0}}e^{-(d+h)\tau }\\ \text{0} & \text{0} & \text{0}\\ \text{0} & \text{0} & \text{0} \end{array}). \end{array}$$

$$\begin{array}{c} V=DV\left(\varepsilon_{\text{0}}\right)=(\begin{array}{ccc} a_{E} & \text{0} & \text{0}\\ -\alpha p_{\text{1}} & a_{A} & \text{0}\\ -\alpha (\text{1}-p_{\text{1}}) & -\delta (\text{1}-p_{\text{2}}) & a_{C} \end{array}). \end{array}$$

Because V is lower triangular with positive diagonal entries, it is nonsingular. Its inverse is


$$\begin{array}{c} V^{-\text{1}}=(\begin{array}{ccc} \frac{\text{1}}{a_{E}} & \text{0} & \text{0}\\ \frac{\alpha p_{\text{1}}}{a_{E}a_{A}} & \frac{\text{1}}{a_{A}} & \text{0}\\ \frac{\alpha \left(\text{1}-p_{\text{1}}\right)a_{A}+\alpha p_{\text{1}}\delta \left(\text{1}-p_{\text{2}}\right)}{\alpha_{E}a_{A}a_{C}}\; & \frac{\delta \left(\text{1}-p_{\text{2}}\right)}{a_{A}a_{C}} & \frac{\text{1}}{a_{C}} \end{array}). \end{array}$$

4. Calculation of the spectral radius

The next-generation matrix is $K=FV^{-\text{1}}$. Since only the first row of F is nonzero, K has two zero rows and can be written as


$$\begin{array}{c} K=FV^{-\text{1}}=(\begin{array}{ccc} R_{\text{0}} & S_{\text{0}}e^{-(d+h)\tau \left[\frac{\beta_{A}}{a_{A}}\;+\;\frac{\beta_{C}\delta \left(\text{1}-p_{\text{2}}\right)}{a_{A}a_{C}}\right]} & \frac{\beta_{C}S_{\text{0}}e^{-}(d+h)\tau }{a_{C}}\\ \text{0} & \text{0} & \text{0}\\ \text{0} & \text{0} & \text{0} \end{array}). \end{array}$$

The eigenvalues of K are 0, 0, and its first diagonal entry. Consequently, the spectral radius ρ(K) equals that nonzero eigenvalue. Therefore,


$$\begin{array}{c} R_{\text{0}}=S_{\text{0}}e^{-(d+h)\tau }\left[\frac{\beta_{A}\alpha p_{\text{1}}}{a_{E}a_{A}}+\frac{\beta_{C}\alpha \left(\text{1}-p_{\text{1}}\right)}{a_{E}a_{C}}+\frac{\beta_{C}\alpha p_{\text{1}}\delta \left(\text{1}-p_{\text{2}}\right)}{a_{E}a_{A}a_{C}}\right]. \end{array}$$

### 2.4. Sensitivity analysis

To identify the parameters that most strongly influence disease invasion, the normalized forward sensitivity index of the basic reproduction number is defined, for any parameter p, by $S_{p}^{R_{\text{0}}}=\frac{\partial R_{\text{0}}}{\partial p}\frac{p}{R_{\text{0}}}$.

A positive index indicates that increasing the parameter increases $R_{\text{0}},$ whereas a negative index indicates a suppressive effect. Let

m=d+h, $a_{E}=m+\alpha$, $a_{A}=m+\mu_{A}+\delta$, $a_{C}=\mu_{C}+\gamma$,

$Q_{A}=\frac{\beta_{A}p_{\text{1}}}{a_{A}}$, $Q_{C}=\frac{\beta_{C}(\text{1}-p_{\text{1}})}{a_{C}}$, $a_{AC}=\frac{\beta_{C}p_{\text{1}}\delta (\text{1}-p_{\text{2}})}{a_{A}a_{C}}$, $Q=Q_{A}+Q_{C}+Q_{AC}$.

Using the expression for $R_{\text{0}}$ derived in Section 2.3, the principal indices are

$S_{\Lambda }^{R_{\text{0}}}=\text{1}$, $S_{\alpha }^{R_{\text{0}}}=\frac{m}{a_{E}}$, $S_{\tau }^{R_{\text{0}}}=-m\tau$,

$S_{\beta_{A}}^{R_{\text{0}}}=\frac{Q_{A}}{Q}$, $S_{\beta_{C}}^{R_{\text{0}}}=\frac{Q_{C}+Q_{AC}}{Q}$,

$S_{\mu_{A}}^{R_{\text{0}}}=-\frac{\mu_{A}}{a_{A}}\frac{Q_{A}+Q_{AC}}{Q}$, $S_{\gamma }^{R_{\text{0}}}=-\frac{\gamma }{a_{C}}$ $\frac{Q_{C}+Q_{AC}}{Q}$, $S_{\mu_{C}}^{R_{\text{0}}}=-\frac{\mu_{C}}{a_{C}}$ $\frac{Q_{C}+Q_{AC}}{Q}$.

Thus, recruitment, progression to infectiousness, and the acute and chronic transmission coefficients increase $R_{\text{0}}$, whereas a longer delay, faster recovery, and greater disease-induced removal reduce it. Since $p_{\text{1}},\;p_{\text{2}}$ and δ affect competing pathways, their indices are evaluated from the complete $R_{\text{0}}$ expression at the baseline parameter set. Parameters are ranked according to $\left|S_{p}^{R_{\text{0}}}\right|$, and the most influential parameters are varied in the simulations.

## 3. Stability analysis

For the stability analysis, we examine two categories of stability: local and global, determined through the equilibrium points. A mathematical stability analysis is carried out separately at the disease-free and endemic equilibrium points to improve the mathematical rigour of the stability analysis. Local stability (asymptotic stability) is determined from the characteristic polynomial using the Routh–Hurwitz criterion. It can be seen that the disease-free equilibrium is locally asymptotically stable when $R_{\text{0}}<\text{1}$, since all the eigenvalues of the linearized system are then negative. The characteristic polynomial of the local equilibrium (0,0) has positive coefficients and the Routh-Hurwitz determinants are positive, resulting in the local equilibrium being locally asymptotically stable. A proper positive-definite Lyapunov function (L) is constructed for each equilibrium point to ensure global stability of the system. The time derivative of it is easily reduced along the solutions of the model according to the relations of the equilibrium and the threshold condition on $R_{\text{0}}$. At the given conditions, $\dot{L}\leq \text{0}$, and the equality is only valid at the corresponding equilibrium point. Thus, the invariant subset of the largest dimension for $\dot{L}=\text{0}$ is only the equilibrium. This invariance principle then implies the global asymptotic stability of the equilibrium for the system of La Salle.

***lemma 3:*** The system (1)-(5) is locally asymptotically stable at the equilibrium point


$$\begin{array}{c} C_{\text{1}}=\left(S^{\text{1}},E^{\text{1}}{,I_{A}}^{\text{1}}{,I_{C}}^{\text{1}},\;R^{\text{1}}\right)=\left(\frac{\Lambda }{d+h},\text{0},\text{0},\text{0},\text{0}\right) \end{array}$$

Provided that $R_{\text{0}}<\text{1}$.

***Proof:*** By evaluating the Jacobian matrix of system (1)–(5), we derived the following form of the system:


$$\begin{array}{c} J=[\begin{array}{ccc} -(\text{d}+\text{h})-\left(\beta_{\text{A}}{\text{I}}_{\text{A}}+\beta_{\text{C}}{\text{I}}_{\text{C}}\right){\text{e}}^{-(\text{d}+\text{h})\tau } & \begin{array}{ccc} \text{0} & \;{-\beta }_{\text{A}}\text{S}{\text{e}}^{-(\text{d}+\text{h})\tau } & {-\beta }_{\text{C}}\text{S}{\text{e}}^{-(\text{d}+\text{h})\tau } \end{array} & \nu \\ \left(\beta_{\text{A}}{\text{I}}_{\text{A}}+\beta_{\text{C}}{\text{I}}_{\text{C}}\right){\text{e}}^{-(\text{d}+\text{h})\tau } & \begin{array}{ccc} -(\text{d}+\text{h}+\alpha )\; & {-\beta }_{\text{A}}\text{S}{\text{e}}^{-(\text{d}+\text{h})\tau } & {-\beta }_{\text{C}}\text{S}{\text{e}}^{-(\text{d}+\text{h})\tau } \end{array} & \text{0}\\ \begin{array}{c} \text{0}\\ \text{0}\\ \text{0} \end{array} & \begin{array}{ccc} \begin{array}{c} \alpha {\text{p}}_{\text{1}}\\ \alpha ({\text{1}-\text{p}}_{\text{1}})\\ \text{0} \end{array} & \begin{array}{c} \left(\text{d}+\text{h}+\mu_{\text{A}}+\delta \right)\\ \delta (\text{1}-{\text{p}}_{\text{2}})\\ \delta {\text{p}}_{\text{1}} \end{array} & \begin{array}{c} \text{0}\\ -(\text{d}+\text{h}+\mu_{\text{C}}+\gamma )\\ \gamma \end{array} \end{array} & \begin{array}{c} \text{0}\\ \text{0}\\ -(\text{d}+\text{h}+\nu ) \end{array} \end{array}]. \end{array}$$

For point$\;C_{\text{1}}=\left(\frac{\Lambda }{d+h},\text{0},\text{0},\text{0},\text{0}\right),$ we have


$$\begin{array}{c} J=[\begin{array}{ccccc} -(d+h) & \text{0} & {-\beta }_{\text{A}}(\frac{\Lambda }{d+h}){\text{e}}^{-(\text{d}+\text{h})\tau } & {-\beta }_{\text{C}}(\frac{\Lambda }{d+h}){\text{e}}^{-(\text{d}+\text{h})\tau } & \nu \\ \text{0} & -(\text{d}+\text{h}+\alpha ) & {-\beta }_{\text{A}}(\frac{\Lambda }{d+h}){\text{e}}^{-(\text{d}+\text{h})\tau } & {-\beta }_{\text{C}}(\frac{\Lambda }{d+h}){\text{e}}^{-(\text{d}+\text{h})\tau } & \text{0}\\ \text{0} & \alpha {\text{p}}_{\text{1}} & \left(\text{d}+\text{h}+\mu_{\text{A}}+\delta \right) & \text{0} & \text{0}\\ \text{0} & \alpha ({\text{1}-\text{p}}_{\text{1}}) & \delta (\text{1}-{\text{p}}_{\text{2}}) & -(\text{d}+\text{h}+\mu_{\text{C}}+\gamma ) & \text{0}\\ \text{0} & \text{0} & \delta {\text{p}}_{\text{1}} & \gamma & -(\text{d}+\text{h}+\nu ) \end{array}]. \end{array}$$

Now, consider


$$\begin{array}{c} |J-\lambda I|=\text{0}. \end{array}$$

So,


$$\begin{array}{c} J-\lambda I=\left|\begin{array}{ccccc} -(d+h)-\lambda & \text{0} & {-\beta }_{\text{A}}\left(\frac{\Lambda }{d+h}\right){\text{e}}^{-(\text{d}+\text{h})\tau } & {-\beta }_{\text{C}}\left(\frac{\Lambda }{\text{d}+\text{h}}\right){\text{e}}^{-(\text{d}+\text{h})\tau } & \nu \\ \text{0} & -(\text{d}+\text{h}+\alpha )-\lambda & {-\beta }_{\text{A}}\left(\frac{\Lambda }{\text{d}+\text{h}}\right){\text{e}}^{-(\text{d}+\text{h})\tau } & {-\beta }_{\text{C}}\left(\frac{\Lambda }{\text{d}+\text{h}}\right){\text{e}}^{-(\text{d}+\text{h})\tau } & \text{0}\\ \text{0} & \alpha {\text{p}}_{\text{1}} & -\left(\text{d}+\text{h}+\mu_{\text{A}}+\delta \right)-\lambda & \text{0} & \text{0}\\ \text{0} & \alpha ({\text{1}-\text{p}}_{\text{1}}) & \delta (\text{1}-{\text{p}}_{\text{2}}) & -\left(\text{d}+\text{h}+\mu_{\text{C}}+\gamma \right)-\lambda & \text{0}\\ \text{0} & \text{0} & \delta {\text{p}}_{\text{1}} & \gamma & -(\text{d}+\text{h}+\nu )-\lambda \end{array}\right| \end{array}$$

It gives the values ofλ


$$\begin{array}{c} \lambda_{\text{1}}=-(d+h)<\text{0}, \end{array}$$

$$\begin{array}{c} \lambda_{\text{2}}=\;-(d+h+\alpha )<\text{0}, \end{array}$$

$$\begin{array}{c} \lambda_{\text{3}}=\;-\left(d+h+\mu_{A}+\delta \right)<\text{0}, \end{array}$$

$$\begin{array}{c} \lambda_{\text{4}}=-\left(d+h+\mu_{C}+\gamma \right)<\text{0}, \end{array}$$

$$\begin{array}{c} \lambda_{\text{5}}=-(d+h+\nu )<\text{0}. \end{array}$$

It shows that $C_{\text{1}}$ is asymptotically stable, $R_{\text{0}}<\text{1}$.

***Lemma 4***: The system (1)-(5) is locally asymptotically stable at $C_{\text{2}}=\left(S^{*},E^{*}{,I_{A}}^{*}{,I_{C}}^{*},R^{*}\right)$ if $R_{\text{0}}>\text{1}$.

Proof: For finding the eigenvalues at $C_{\text{2}}=\left(S^{*},E^{*},{I_{A}}^{*}{,I_{C}}^{*},R^{*}\right)\;$by using the Jacobian matrix


$$\begin{array}{c} J=\left|\begin{array}{ccccc} A_{\text{1}}-\lambda & \text{0} & A_{\text{2}} & A_{\text{3}} & \nu \\ A_{\text{4}} & A_{\text{5}}-\lambda & A_{\text{6}} & A_{\text{7}} & \text{0}\\ \text{0} & A_{\text{8}} & A_{\text{9}}-\lambda & \text{0} & \text{0}\\ \text{0} & A_{\text{1}\text{0}} & A_{\text{1}\text{1}} & A_{\text{1}\text{2}}-\lambda & \text{0}\\ \text{0} & \text{0} & A_{\text{1}\text{3}} & A_{\text{1}\text{4}} & A_{\text{1}\text{5}}-\lambda \end{array}\right|, \end{array}$$

$$\begin{array}{c} {=\lambda }^{\text{5}}+\lambda^{\text{4}}B+\lambda^{\text{3}}C+\lambda^{\text{2}}D+\lambda E+F. \end{array}$$

According to the Routh–Hurwitz criterion, when $R_{\text{0}}>\text{1}$and the coefficients B,C,D,E, and F are positive, the equilibrium point $C_{\text{2}}$ is locally asymptotically stable.

***Lemma 5***: To find the global stability at $C_{\text{1}}$. The system (1)-(5) is GAS at $C_{\text{1}}=\left(S^{\text{1}},E^{\text{1}}{,I_{A}}^{\text{1}}{,I_{C}}^{\text{1}},\;R^{\text{1}}\right)=\left(\frac{\Lambda }{d+h},\text{0},\text{0},\text{0},\text{0}\right)$ if$R_{\text{0}}\;>\text{1}.$

Proof: To prove the theorem, we use the Lyapunov function U:Ω→R defined as


$$\begin{array}{c} U\left(I_{A}\right)=\text{ln}\left(\frac{I_{A}}{I_{A_{\text{0}}}}\;\right), \end{array}$$

$$\begin{array}{c} \frac{dU\left(I_{A}\right)}{dt}=\;\frac{\text{1}}{I_{A}}\left[\alpha p_{\text{1}}\;E(t)-\left(d+h+\mu_{A}+\delta \right)I_{A}(t)\right], \end{array}$$

$$\begin{array}{c} \frac{dU\left(I_{A}\right)}{dt}=\left[\frac{\alpha p_{\text{1}}E}{I_{A}}-\left(d+h+\mu_{A}+\delta \right)\right], \end{array}$$

$$\begin{array}{c} \frac{dU\left(I_{A}\right)}{dt}\leq \left(d+h+\mu_{A}+\delta \right)[\text{0}-\text{1}], \end{array}$$

$$\begin{array}{c} \frac{dU\left(I_{A}\right)}{dt}\;\leq \text{0}. \end{array}$$

and


$$\begin{array}{c} U\left(I_{C}\right)=\text{ln}\left(\frac{I_{C}}{I_{C_{\text{0}}}}\;\right), \end{array}$$

$$\begin{array}{c} \frac{dU\left(I_{C}\right)}{dt}=\;\frac{\text{1}}{I_{c}}\left[\alpha \left(\text{1}-p_{\text{1}}\right)E(t)+\delta \left(\text{1}-p_{\text{2}}\right)I_{A}(t)-\left(d+h+\mu_{C}+\gamma \right)I_{C}(t)\right], \end{array}$$

$$\begin{array}{c} \frac{dU\left(I_{C}\right)}{dt}=\left[\frac{\alpha \left(\text{1}-p_{\text{1}}\right)E(t)}{I_{c}}+\frac{\delta \left(\text{1}-p_{\text{2}}\right)I_{A}(t)}{I_{c}}-\left(d+h+\mu_{C}+\gamma \right)\right], \end{array}$$

$$\begin{array}{c} \frac{dU\left(I_{C}\right)}{dt}\leq \;\left(d+h+\mu_{C}+\gamma \right)[\text{0}-\text{0}-\text{1}], \end{array}$$

So,

$\frac{dU\left(I_{C}\right)}{dt}\leq \text{0}$ if $R_{\text{0}}\;<\text{1}.$

As desired.

***Lemma 6:*** For the global asymptotically stable of system (1)-(5) at $C_{\text{2}}=\left(S^{*},E^{*}{,I_{A}}^{*}{,I_{C}}^{*},\;R^{*}\right)$, if $R_{\text{0}}\;>\text{1}$.

Proof: Firstly, we put right hand side of the system (1)–(5) is equal to zero:


$$\begin{array}{c} \Lambda -(d+h)S(t)-\left(\beta_{A}SI_{A}+\beta_{C}SI_{C}\right)e^{-(d+h)\tau }+\nu R(t)=\text{0}, \end{array}$$

$$\begin{array}{c} \left(\beta_{A}SI_{A}+\beta_{C}SI_{C}\right)e^{-(d+h)\tau }-(d+h+\alpha )E(t)=\text{0}, \end{array}$$

$$\begin{array}{c} \alpha p_{\text{1}}E(t)-\left(d+h+\mu_{A}+\delta \right)I_{A}(t)=\text{0}, \end{array}$$

$$\begin{array}{c} \alpha \left(\text{1}-p_{\text{1}}\right)E(t)+\delta \left(\text{1}-p_{\text{2}}\right)I_{A}(t)-\left(d+h+\mu_{C}+\gamma \right)I_{C}(t)=\text{0}, \end{array}$$

$$\begin{array}{c} \delta p_{\text{2}}I_{A}+\gamma I_{C}-(d+h+\nu )R(t)=\text{0}. \end{array}$$

By Lyapunov function, Z:Ω→R defined as


$$\begin{array}{cc} Z=\; & K_{\text{1}}\left(S-S^{*}-S^{*}\frac{logS^{*}}{S^{*}}\right)+K_{\text{1}}\left(E-E^{*}-E^{*}\frac{logE^{*}}{E^{*}}\right)+K_{\text{1}}\left(I_{A}-{I_{A}}^{*}-{I_{A}}^{*}\frac{log{I_{A}}^{*}}{{I_{A}}^{*}}\right)+K_{\text{1}}\left(I_{C}-{I_{C}}^{*}-{I_{C}}^{*}\frac{log{I_{C}}^{*}}{{I_{C}}^{*}}\right)\\ & \;\;+K_{\text{1}}\left(R-R^{*}-R^{*}\frac{logR^{*}}{R^{*}}\right), \end{array}$$

$$\begin{array}{c} \frac{dZ}{dt}=K_{\text{1}}\left(\frac{S-S^{*}}{S}\right)\frac{dS}{dt}+K_{\text{2}}\left(\frac{E-E^{*}}{E}\right)\frac{dE}{dt}+K_{\text{3}}\left(\frac{I_{A}-{I_{A}}^{*}}{I_{A}}\right)\frac{dI_{A}}{dt}+K_{\text{4}}\left(\frac{I_{C}-{I_{C}}^{*}}{I_{C}}\right)\frac{dI_{C}}{dt}+K_{\text{5}}\left(\frac{R-R^{*}}{R}\right)\frac{dR}{dt}, \end{array}$$

$$\begin{array}{cc} = & \left(-\frac{\Lambda }{SS^{*}}{\left(S-S^{*}\right)}^{\text{2}}-\frac{\alpha \text{R}}{SS^{*}}{\left(S-S^{*}\right)}^{\text{2}}\right)+\left(-\frac{\beta_{A}SI_{A}e^{-(d+h)\tau }}{EE^{*}}{\left(E-E^{*}\right)}^{\text{2}}-\frac{\beta_{C}SI_{C}e^{-(d+h)\tau }}{EE^{*}}{\left(E-E^{*}\right)}^{\text{2}}\right)\\ & +\left(-\frac{\alpha p_{\text{1}}E}{I_{A}{I_{A}}^{*}}{\left(I_{A}-{I_{A}}^{*}\right)}^{\text{2}}\right)+\left(-\frac{\alpha \left(\text{1}-p_{\text{1}}\right)E}{I_{C}{I_{C}}^{*}}{\left(I_{C}-{I_{C}}^{*}\right)}^{\text{2}}-\frac{\delta \left(\text{1}-p_{\text{2}}\right)I_{A}}{I_{C}-{I_{C}}^{*}}{\left(I_{C}-{I_{C}}^{*}\right)}^{\text{2}}\right)\\ & +\left(-\frac{\delta p_{\text{2}}I_{A}}{RR^{*}}{\left(R-R^{*}\right)}^{\text{2}}-\frac{\gamma I_{C}}{RR^{*}}{\left(R-R^{*}\right)}^{\text{2}}\right), \end{array}$$

$$\begin{array}{c} =-\frac{\Lambda }{SS^{*}}{\left(S-S^{*}\right)}^{\text{2}}-\frac{\alpha \text{R}}{SS^{*}}{\left(S-S^{*}\right)}^{\text{2}}<\text{0}, \end{array}$$

$-\frac{\beta_{A}SI_{A}e^{-(d+h)\tau }}{EE^{*}}{\left(E-E^{*}\right)}^{\text{2}}-\frac{\beta_{C}SI_{C}e^{-(d+h)\tau }}{EE^{*}}{\left(E-E^{*}\right)}^{\text{2}}<\text{0}$,

$-\frac{\alpha p_{\text{1}}E}{I_{A}{I_{A}}^{*}}{\left(I_{A}-{I_{A}}^{*}\right)}^{\text{2}}<\text{0}$,

$-\frac{\alpha \left(\text{1}-p_{\text{1}}\right)E}{I_{C}{I_{C}}^{*}}{\left(I_{C}-{I_{C}}^{*}\right)}^{\text{2}}-\frac{\delta \left(\text{1}-p_{\text{2}}\right)I_{A}}{I_{C}-{I_{C}}^{*}}{\left(I_{C}-{I_{C}}^{*}\right)}^{\text{2}}<\text{0}$ *,*

$-\frac{\delta p_{\text{2}}I_{A}}{RR^{*}}{\left(R-R^{*}\right)}^{\text{2}}-\frac{\gamma I_{C}}{RR^{*}}{\left(R-R^{*}\right)}^{\text{2}}<\text{0}$.

## 4. Transition probabilities

The number of potential outcomes is displayed in Table 2 as follows.

**Table 2:** Transition Probabilities of the model.

$T_{i}=Transitions$	$P_{i}=Probabilities$
$T_{1}={[1,0,0,0,0]}^{T}$	$P_{\text{1}}=(\Lambda )\Delta T$
$T_{2}={[-1,0,0,0,0]}^{T}$	$P_{\text{2}}=(\text{d}+\text{h})\text{S}\Delta T$
$T_{3}={[-1,1,0,0,0]}^{T}$	$P_{\text{3}}={\beta_{A}S{I_{A}}^{*}e}^{-(d+h)\tau }\Delta T$
$T_{4}={[-1,1,0,0,0]}^{T}$	$P_{\text{4}}={\beta_{C}S{I_{C}}^{*}e}^{-(d+h)\tau }\Delta T$
$T_{5}={[1,0,0,0,-1]}^{T}$	$P_{\text{5}}=\nu \text{R}\Delta T$
$T_{6}={[0,-1,0,0,0]}^{T}$	$P_{\text{6}}=(\text{d}+\text{h})\text{E}\Delta T$
$T_{7}={[0,-1,0,1,0]}^{T}$	$P_{\text{7}}=\alpha \text{E}\Delta T$
$T_{8}={[0,0,1,-1,0]}^{T}$	$P_{\text{8}}=\alpha p_{\text{1}}\text{E}\Delta T$
$T_{9}={[0,0,-1,0,0]}^{T}$	$P_{\text{9}}=(\text{d}+\text{h})I_{A}\Delta T$
$T_{10}={[0,0,-1,0,0]}^{T}$	$P_{\text{1}\text{0}}=\mu_{A}I_{A}\Delta T$
$T_{11}={[0,0,1,1,0]}^{T}$	$P_{\text{1}\text{1}}=\delta I_{A}\Delta T$
$T_{12}={[0,0,0,-1,1]}^{T}$	$P_{\text{1}\text{2}}={\delta p}_{\text{2}}I_{A}\Delta T$
$T_{13}={[0,0,0,-1,0]}^{T}$	$P_{\text{1}\text{3}}=(\text{d}+\text{h})I_{C}\Delta T$
$T_{14}={[0,0,0,-1,0]}^{T}$	$P_{\text{1}\text{4}}=\mu_{C}I_{C}\Delta T$
$T_{15}={[0,0,0,-1,1]}^{T}$	$P_{\text{1}\text{5}}=\gamma I_{C}\Delta T$
$T_{16}={[0,0,0,0,-1]}^{T}$	$P_{\text{1}\text{6}}=(\text{d}+\text{h})\text{R}\Delta T$

Next, we compute the expectation and variance of the drift and diffusion terms of system (1)–(5), as presented below:

**The expected value**


$$\begin{array}{c} {\text{E}}^{*}[\Delta \text{U}]=\sum_{\text{i}=\text{1}}^{\text{7}}{\text{P}}_{\text{i}}{\text{T}}_{\text{i}}=[\begin{array}{c} \Lambda -(d+h)S(t)-\left(\beta_{A}SI_{A}+\beta_{C}SI_{C}\right)e^{-(d+h)\tau }+\nu R(t)\\ \left(\beta_{A}SI_{A}+\beta_{C}SI_{C}\right)e^{-(d+h)\tau }-(d+h+\alpha )E(t)\\ \begin{array}{c} \alpha p_{\text{1}}E(t)-\left(d+h+\mu_{A}+\delta \right)I_{A}\\ \begin{array}{c} \alpha \left(\text{1}-p_{\text{1}}\right)E(t)+\delta \left(\text{1}-p_{\text{2}}\right)I_{A}(t)-\left(d+h+\mu_{C}+\gamma \right)I_{C}(t)\\ \delta p_{\text{2}}I_{A}+\gamma I_{C}-(d+h+\nu )R(t) \end{array} \end{array} \end{array}]\Delta t. \end{array}$$

**Variance**


$$\begin{array}{c} E^{*}\left[\Delta U{\Delta U}^{T}\right]=\sum_{i=\text{1}}^{\text{7}}P_{i}{\left[{\text{T}}_{\text{i}}\right]\left[{\text{T}}_{\text{i}}\right]}^{T} \end{array}$$

$$\begin{array}{c} =[\begin{array}{ccccc} P_{\text{1}}+P_{\text{2}}+P_{\text{3}}+P_{\text{4}}+P_{\text{5}} & -P_{\text{3}}-P_{\text{4}} & \text{0} & \text{0} & -P_{\text{5}}\\ -P_{\text{3}}-P_{\text{4}} & P_{\text{3}}+P_{\text{4}}+P_{\text{6}}+P_{\text{7}} & P_{\text{8}} & {-P}_{\text{7}} & \text{0}\\ \text{0} & \text{0} & P_{\text{8}}+P_{\text{9}}+P_{\text{1}\text{0}}+P_{\text{1}\text{1}} & P_{\text{8}}+P_{\text{1}\text{1}} & \text{0}\\ \text{0} & -P_{\text{7}} & -P_{\text{8}}+P_{\text{1}\text{1}} & P_{\text{8}}+P_{\text{1}\text{1}}+P_{\text{1}\text{2}}+P_{\text{1}\text{3}}+P_{\text{1}\text{4}}+P_{\text{1}\text{5}} & -P_{\text{1}\text{2}}-P_{\text{1}\text{5}}\\ -{\text{P}}_{\text{5}} & \text{0} & \text{0} & -P_{\text{1}\text{2}}-P_{\text{1}\text{5}} & P_{\text{1}\text{2}}+P_{\text{1}\text{5}}+P_{\text{1}\text{6}} \end{array}]\Delta t. \end{array}$$

Drift$=\text{H}\left(\text{U}(\text{t}),\text{t}\right)=\frac{{\text{E}}^{*}[\Delta \text{U}]}{\Delta \text{t}}$,

Diffusion $=\text{K}\left(\text{U}(\text{t}),\text{t}\right)=\sqrt{\frac{{\text{E}}^{*}[\Delta \text{U}{\Delta \text{U}}^{\text{T}}]}{\Delta \text{t}}}$.

So,


$$\begin{array}{c} \text{dU}(\text{t})=\text{H}\left(\text{U}(\text{t}),\text{t}\right)\text{dt}+\text{K}\left(\text{U}(\text{t}),\text{t}\right)\text{dB}(\text{t})\text{.} \end{array}$$ (15)

The above Eq. (15) is called the stochastic differential equation, where B(t) is Brownian motion.

### 4.1. Euler-Maruyama method

In the following section, we discuss the standard numerical methods used to approximate solutions to stochastic models. We will agree that $I_{n}=\{\text{0},\text{1},\text{2},\text{3},\ldots n\}$. A consistent division of the temporal interval [0,T] with uniform partition equal to $\tau =\frac{T}{N}$, N∈N and N the set of natural numbers, is taken into consideration. Their corresponding nodes are given by$\;\text{0}=t_{\text{0}}<t_{\text{1}}<t_{\text{2}}<\ldots t_{N}=T$.

For each $n\in I_{N}$. Further, this will be agreed by us: $U^{n}=U(t_{n})$, however $n\in I_{N}$ and $U(t)={\left(S,I,R\right)}^{t}$. $\Delta W_{n}=W\left(t_{n}+\text{1}\right)-W(t_{n})$, $\Delta W_{n}$ is normal distribution with mean zero and a variance of one [19].

## 5. Stochastic delayed model

Within the modeling of disease transmission, the parameter for time delay signifies the complete incubation period of the pathogen, defined as the time elapsed between the initial infection event and the emergence of symptoms. The introduction of this time delay into the model equations can yield multiple periodic solutions that are dependent on the specific value of τ. Every initial condition can lead to a unique global solution for stochastic differential equations. The system’s coefficients are locally Lipschitz continuous, but they fail to satisfy the linear growth condition. We apply the Lyapunov analysis to the positivity and global system.

The deterministic delayed model describes the average evolution of the Newcastle disease compartments under fixed epidemiological parameters. However, disease transmission is affected by random changes in contact patterns, environmental conditions, host behaviour, and other unobserved factors. Therefore, the deterministic system is extended to a stochastic delay differential model.

Using the transition-probability approach, the conditional mean produces the deterministic drift, while the conditional covariance determines the diffusion component. For the theoretical and numerical analysis, selected parameters are perturbed by multiplicative Brownian noise. Accordingly, the stochastic model may be written as


$$\begin{array}{c} dX(t)=f(X(t),\;X(t-\tau ))dt+H(X(t),\;X(t-\tau ))dW(t), \end{array}$$

where $X(t)=(S(t),\;E(t),\;I_{A}(t)\;,\;I_{C}(t),\;R(t){)}^{T}$, fis the deterministic drift, H contains the noise-intensity terms, and W(t) denotes standard Brownian motion. The multiplicative noise represents random fluctuations whose magnitude depends on the corresponding population compartment. When the noise intensities are zero, the stochastic system reduces exactly to the original deterministic delayed model.

Let cdt=cdt+σdB(t) with parametric perturbation technique for the system (1)-(5) as follows


$$\begin{array}{c} \;\begin{array}{c} d\text{S}(\text{t})=\left[\Lambda -(d+h)S(t)-\left(\beta_{A}SI_{A}+\beta_{C}SI_{C}\right)\;e^{-(d+h)\tau }+\nu R(t)\right]dt+\sigma_{\text{1}}SdB,\\ d\text{E}(\text{t})=\left[\left(\beta_{A}SI_{A}+\beta_{C}SI_{C}\right)e^{-(d+h)\tau }-(d+h+\alpha )E(t)\right]dt+\sigma_{\text{2}}EdB,\\ \begin{array}{c} dI_{A}(\text{t})=\left[\alpha p_{\text{1}}E(t)-\left(d+h+\mu_{A}+\delta \right)I_{A}\right]dt+\sigma_{\text{3}}I_{A}dB,\\ dI_{C}(\text{t})=\left[\alpha \left(\text{1}-p_{\text{1}}\right)E(t)+\delta \left(\text{1}-p_{\text{2}}\right)I_{A}(t)-\left(d+h+\mu_{C}+\gamma \right)I_{C}(t)\right]dt+\sigma_{\text{4}}I_{C}(t)dB,\\ d\text{R}(\text{t})=\left[\delta p_{\text{2}}I_{A}+\gamma I_{C}-(d+h+\nu )R(t)\right]dt+\sigma_{\text{5}}RdB \end{array} \end{array}\} \end{array}$$ (16)

where τ denotes the delay effect, and σ represents randomness, ∀ t≥0. Here, B(t) denotes an independent standard Brownian motion, B(t) represents its stochastic increment, and $\sigma_{i}\geq \text{0}$ is the noise intensity associated with the corresponding compartment. The parameter τ denotes the transmission delay, while X(t−τ) represents the delayed population state. When $\sigma_{i}=\text{0}$ for all i, the stochastic system reduces to the corresponding deterministic delayed model.

### 5.1. Positivity and boundedness

We define a probability space (Ω, F, P) along with a filtration ${\left\{F_{t}\right\}}_{t\epsilon R}$. To ensure that the filtration is increasing and right-continuous [24], the initial $F_{\text{0}}$ is taken to include all P-null sets. Symbolically represented as $U(t)=(S(t),E(t),\;I_{A}(t),I_{C}(t),\;R(t))$ and norm as


$$\begin{array}{c} \left|U(t)\right|=\sqrt{S^{\text{2}}(t)+E^{\text{2}}(t)+{I_{A}}^{\text{2}}(t)+{I_{C}}^{\text{2}}(t)+R^{\text{2}}(t)}. \end{array}$$

***lemma 7***: For any initial conditions $\left(S(\text{0}),E(\text{0}),\;I_{A}(\text{0}),I_{C}(\text{0}),R(\text{0})\right)\in R_{+}^{\text{5}}$, the system described by equation (16) admits a unique solution $\left(S(t),E(t),\;I_{A}(t),I_{C}(t),R(t)\right)$ that exists for all t≥0. Furthermore, this solution remains in the positive region $R_{+}^{\text{5}}$ with probability one.

***Proof***: The model (16), under its initial condition$\left(S(\text{0}),E(\text{0}),\;I_{A}(\text{0}),I_{C}(\text{0}),R(\text{0})\right)\in R_{+}^{\text{5}}$, is locally Lipschitz continuous, which guarantees a unique solution exists on the maximal interval $t\in \left[\text{0},t_{e}\right]$, where $t_{e}$ is the explosion time. Proving that this solution is global and that it persists for all time requires verifying that $t_{e}=\infty$. Assuming $d_{\text{0}}\geq \text{1},$ the initial values are contained within the interval $\left[\frac{\text{1}}{d_{\text{0}}},d_{\text{0}}\right]$. For any integer $d\geq d_{\text{0}}$, this construction defines a stopping time.


$$\begin{array}{c} \tau_{d}=\text{inf}\left\{t\in \left[\text{0},t_{e}\right]:\text{min}\left(S(t),E(t),\;I_{A}(t),I_{C}(t),R(t)\right)\leq \frac{\text{1}}{d}\;or\text{max}\left(S(t),E(t),\;I_{A}(t),I_{C}(t),R(t)\right)\geq d\right\} \end{array}$$ (17)

$\tau_{d}$ is an increasing function as d→∞.

So,


$$\begin{array}{c} \tau_{\infty }={lim}_{d\to \infty }\tau_{d} \end{array}$$ (18)

$$\begin{array}{c} \tau_{\infty }\leq \tau_{e} \end{array}$$

If $\tau_{\infty }=\infty$, then$\;\tau_{e}=\infty$.

The argument’s failure implies the existence of a constant T>0 and a value ε∈(0,1) such that,


$$\begin{array}{c} P\left\{\tau_{\infty }\leq T\right\}>\varepsilon , \end{array}$$ (19)

There exists an integer$\;d_{\text{1}}\geq d_{\text{0}}$.


$$\begin{array}{c} P\left\{\tau_{d}\leq T\right\}>\varepsilon ,\;\forall \;d\geq d_{\text{1}}\text{.} \end{array}$$ (20)

For $t\leq \tau_{d}$. After calculation, we see that

$S(t)+E(t),\;I_{A}(t),I_{C}(t)+R(t)\leq \left\{ \begin{array}{c} \begin{array}{cc} \frac{\Lambda }{\left(d+h\right)}\; & \;if\;S(\text{0})+E(\text{0}),+I_{A}(\text{0}){+I}_{C}(\text{0})+R(\text{0})\leq \frac{\Lambda }{\left(d+h\right)} \end{array}\\ \begin{array}{cc} otherwise & S(\text{0})+E(\text{0})+\;I_{A}(\text{0})+I_{C}(\text{0})+R(\text{0})\geq \frac{\Lambda }{\left(d+h\right)} \end{array} \end{array}\right.\;$.

Define a $C^{\text{5}}$-function $V:R_{+}^{\text{5}}\to R_{+}$ by


$$\begin{array}{c} V\left(S,E,I_{A},I_{C},R\right)=(S-\text{1}-lnS)+(E-\text{1}-lnE)+\left(I_{A}-\text{1}-lnI_{A}\right)+\left(I_{C}-\text{1}-lnI_{C}\right)+(R-\text{1}-lnR)\text{.} \end{array}$$ (21)

From Ito’s formula, we can get

$dV\left(S,E,I_{A},I_{C},R\right)=\left(\text{1}-\frac{\text{1}}{S}\right)dS+\left(\text{1}-\frac{\text{1}}{E}\right)dE+\left(\text{1}-\frac{\text{1}}{I_{A}}\right)dI_{A}+\left(\text{1}-\frac{\text{1}}{I_{C}}\right)dI_{C}+\left(\text{1}-\frac{\text{1}}{R}\right)dR+\frac{\sigma^{\text{2}}}{\text{2}}dt$.

Putting values ofdS,dI,dR.

$=\left[\Lambda -(d+h)\left(S+E+I_{A}+I_{C}+R\right)-\mu_{A}I_{A}-\mu_{C}I_{C}+\frac{\sigma^{\text{2}}}{\text{2}}\right]dt+\sigma \left(S+E+I_{A}+I_{C}\right)dB(t)$.

Let $Z=\Lambda -(d+h)\left(S+E+I_{A}+I_{C}+R\right)-\mu_{A}I_{A}-\mu_{C}I_{C}+\frac{\sigma^{\text{2}}}{\text{2}}$, where Z is a constant. The above equation is


$$\begin{array}{c} dV\left(S,E,I_{A},I_{C},R\right)\leq Zdt+\sigma \left(S+E+I_{A}+I_{C}\right)dB(t). \end{array}$$ (22)

After integration from $\text{0}\;to\;\tau_{d}\wedge \tau ,$ where $\tau_{d}\wedge \tau =\text{min}\left(\tau_{d},T\right).$ Therefore, taking expectations. We get


$$\begin{array}{c} EV\left(S\left(\tau_{d}\wedge \tau \right),E\left(\tau_{d}\wedge \tau \right),I\left(\tau_{d}\wedge \tau \right),I\left(\tau_{d}\wedge \tau \right),R\left(\tau_{d}\wedge \tau \right)\right)\leq V\left(S(\text{0}),E(\text{0}),I_{A}(\text{0}),I_{C}(\text{0}),R(\text{0})\right)+ZT\text{.} \end{array}$$ (23)

Let,


$$\begin{array}{c} \Omega_{d}=\left\{\tau_{d}\leq \tau \right\}. \end{array}$$

For every, $g\;\epsilon \;\Omega_{d}$. For some $j,\;h_{j}(\tau_{d},g)$ equals either d or$\;\frac{\text{1}}{d}$ for j = 1,2,3.

Hence,


$$\begin{array}{c} V(S\left(\tau_{d},u\right),E\left(\tau_{d}\wedge \tau \right),I_{A}\left(\tau_{d}\wedge \tau \right),I_{C}\left(\tau_{d}\wedge \tau \right),R\left(\tau_{d},u\right)<\text{min}\left\{d-\text{1}-lnd,\;\frac{\text{1}}{d}-\text{1}-ln\frac{\text{1}}{d}\right\}, \end{array}$$

obtained that result


$$\begin{array}{cc} & V\left(S(\text{0}),E(\text{0}),\;I_{A}(\text{0}),I_{C}(\text{0}),R(\text{0})\right)+ZT\geq E[{I_{A}}_{\Omega_{d}g}V\left(S\left(\tau_{d},u\right),E\left(\tau_{d}\wedge \tau \right),I_{A}\left(\tau_{d}\wedge \tau \right),I_{C}\left(\tau_{d}\wedge \tau \right),R\left(\tau_{d},u\right)\right)\\ & +{I_{A}}_{\Omega_{d}g}V\left(S\left(\tau_{d},u\right),E\left(\tau_{d}\wedge \tau \right),I_{A}\left(\tau_{d}\wedge \tau \right),I_{C}\left(\tau_{d}\wedge \tau \right),R\left(\tau_{d},u\right)\right)]\;\geq \varepsilon [(d-\text{1}-lnd)\wedge (\frac{\text{1}}{d}-\text{1}-ln\frac{\text{1}}{d}\;)]\text{.} \end{array}$$ (24)

For d→∞ leads to contradiction $V\left(S(\text{0}),E(\text{0}),I_{A}(\text{0}),I_{C}(\text{0}),R(\text{0})\right)+ZT<\infty$ as desired.

## 6. Numerical analysis

This section evaluates both conventional and non-standard numerical methods for simulating the stochastic model, along with their respective results. The non-standard approach, pioneered by R. E. Mickens, is especially notable for achieving high accuracy with low computational expense. Furthermore, this technique preserves key qualitative properties inherent to the exact solutions.

### 6.1. Stochastic Euler method


$$\begin{array}{c} S^{n+\text{1}}=S^{n}+h\left[\;\Lambda -(d+h)S^{n}-\left(\beta_{A}S^{n}{I_{A}}^{n}+\beta_{C}S^{n}{I_{C}}^{n}\right)e^{-(d+h)\tau }+\nu R^{n}(t)\right]dt+\sigma_{\text{1}}S^{n}\Delta B_{n}, \end{array}$$

$$\begin{array}{c} E^{n+\text{1}}=E^{n}+h\left[\;\left(\beta_{A}S^{n}{I_{A}}^{n}+\beta_{C}S^{n}{I_{C}}^{n}\right)e^{-(d+h)\tau }-(d+h+\alpha )E^{n}(t)\right]dt+\sigma_{\text{2}}E^{n}\Delta B_{n}, \end{array}$$

$$\begin{array}{c} {I_{A}}^{n+\text{1}}={I_{A}}^{n}+h\left[\alpha p_{\text{1}}E^{n}(t)-\left(d+h+\mu_{A}+\delta \right){I_{A}}^{n}\right]dt+\sigma_{\text{3}}{I_{A}}^{n}\Delta B_{n}, \end{array}$$

$$\begin{array}{c} {I_{C}}^{n+\text{1}}={I_{C}}^{n}+h\left[\alpha \left(\text{1}-p_{\text{1}}\right)E^{n}(t)+\delta \left(\text{1}-p_{\text{2}}\right){I_{A}}^{n}(t)-\left(d+h+\mu_{C}+\gamma \right){I_{C}}^{n}(t)\right]dt+\sigma_{\text{4}}{I_{C}}^{n}\Delta B_{n}, \end{array}$$

$$\begin{array}{c} R^{n+\text{1}}=R^{n}+h\left[\delta p_{\text{2}}{I_{A}}^{n}+\gamma {I_{C}}^{n}-(d+h+\nu )R^{n}(t)\right]dt+\sigma_{\text{5}}R^{n}\Delta B_{n}. \end{array}$$

### 6.2. Stochastic Runge-Kutta method

**Stage 1**

$\begin{array}{c} L_{1}=\;h\left[\;\Lambda -(d+h)S^{n}-\left(\beta_{A}S^{n}{I_{A}}^{n}+\beta_{C}S^{n}{I_{C}}^{n}\right)e^{-(d+h)\tau }+\nu R^{n}(t)\right]dt+\sigma_{\text{1}}S^{n}\Delta B_{n}, \end{array}$

$M_{1}=\;h\left[\;\left(\beta_{A}S^{n}{I_{A}}^{n}+\beta_{C}S^{n}{I_{C}}^{n}\right)e^{-(d+h)\tau }-(d+h+\alpha )E^{n}(t)\right]dt+\sigma_{\text{2}}E^{n}\Delta B_{n},$

$N_{1}=h\left[\alpha p_{\text{1}}E^{n}(t)-\left(d+h+\mu_{A}+\delta \right){I_{A}}^{n}\right]dt+\sigma_{\text{3}}{I_{A}}^{n}\Delta B_{n},$

$O_{1}=\;h\left[\alpha \left(\text{1}-p_{\text{1}}\right)E^{n}(t)+\delta \left(\text{1}-p_{\text{2}}\right){I_{A}}^{n}(t)-\left(d+h+\mu_{C}+\gamma \right){I_{C}}^{n}(t)\right]dt+\sigma_{\text{4}}{I_{C}}^{n}\Delta B_{n},$

$Q_{1}=\;h\left[\delta p_{\text{2}}{I_{A}}^{n}+\gamma {I_{C}}^{n}-(d+h+\nu )R^{n}(t)\right]dt+\sigma_{\text{5}}R^{n}\Delta B_{n}.$

**Stage 2**

$\begin{array}{c} L_{2}=\;h\left[\;\Lambda -(d+h){(S}^{n}+\frac{L_{1}}{\text{2}})-\left(\beta_{A}{(S}^{n}+\frac{L_{1}}{\text{2}})({I_{A}}^{n}+\frac{N_{1}}{\text{2}})+\beta_{C}{(S}^{n}+\frac{L_{1}}{\text{2}})({I_{C}}^{n}+\frac{O_{1}}{\text{2}})\right)e^{-(d+h)\tau }+\nu {(R}^{n}+\frac{Q_{1}}{\text{2}})\right]dt+\sigma_{\text{1}}{(S}^{n}+\frac{L_{1}}{\text{2}})\Delta B_{n}, \end{array}$

$M_{2}=\;h\left[\;\left(\beta_{A}{(S}^{n}+\frac{L_{1}}{\text{2}})({I_{A}}^{n}+\frac{N_{1}}{\text{2}})+\beta_{C}{(S}^{n}+\frac{L_{1}}{\text{2}})({I_{C}}^{n}+\frac{O_{1}}{\text{2}})\right)e^{-(d+h)\tau }-(d+h+\alpha ){(E}^{n}+\frac{M_{1}}{\text{2}})\right]dt+\sigma_{\text{2}}{(E}^{n}+\frac{M_{1}}{\text{2}})\Delta B_{n},$

$N_{2}=h\left[\alpha p_{\text{1}}{(E}^{n}+\frac{M_{1}}{\text{2}})-\left(d+h+\mu_{A}+\delta \right)({I_{A}}^{n}+\frac{N_{1}}{\text{2}})\right]dt+\sigma_{\text{3}}({I_{A}}^{n}+\frac{N_{1}}{\text{2}})\Delta B_{n},$

$O_{2}=\;h\left[\alpha \left(\text{1}-p_{\text{1}}\right){(E}^{n}+\frac{M_{1}}{\text{2}})+\delta \left(\text{1}-p_{\text{2}}\right)({I_{A}}^{n}+\frac{N_{1}}{\text{2}})-\left(d+h+\mu_{C}+\gamma \right)({I_{C}}^{n}+\frac{O_{1}}{\text{2}})\right]dt+\sigma_{\text{4}}({I_{C}}^{n}+\frac{O_{1}}{\text{2}})\Delta B_{n},$

$Q_{2}=\;h\left[\delta p_{\text{2}}({I_{A}}^{n}+\frac{N_{1}}{\text{2}})+\gamma ({I_{C}}^{n}+\frac{O_{1}}{\text{2}})-(d+h+\nu ){(R}^{n}+\frac{Q_{1}}{\text{2}})\right]dt+\sigma_{\text{5}}{(R}^{n}+\frac{Q_{1}}{\text{2}})\Delta B_{n}.$

**Stage 3**

$\begin{array}{c} L_{3\;}=\;h\left[\;\Lambda -(d+h){(S}^{n}+\frac{L_{2}}{\text{2}})-\left(\beta_{A}{(S}^{n}+\frac{L_{2}}{\text{2}})({I_{A}}^{n}+\frac{N_{2}}{\text{2}})+\beta_{C}{(S}^{n}+\frac{L_{2}}{\text{2}})({I_{C}}^{n}+\frac{O_{2}}{\text{2}})\right)e^{-(d+h)\tau }+\nu {(R}^{n}+\frac{Q_{2}}{\text{2}})\right]dt+\sigma_{\text{1}}{(S}^{n}+\frac{L_{1}}{\text{2}})\Delta B_{n}, \end{array}$

$M_{3}=\;h\left[\;\left(\beta_{A}{(S}^{n}+\frac{L_{2}}{\text{2}})({I_{A}}^{n}+\frac{N_{2}}{\text{2}})+\beta_{C}{(S}^{n}+\frac{L_{2}}{\text{2}})({I_{C}}^{n}+\frac{O_{2}}{\text{2}})\right)e^{-(d+h)\tau }-(d+h+\alpha ){(E}^{n}+\frac{M_{2}}{\text{2}})\right]dt+\sigma_{\text{2}}{(E}^{n}+\frac{M_{2}}{\text{2}})\Delta B_{n},$

$N_{3}=h\left[\alpha p_{\text{1}}{(E}^{n}+\frac{M_{2}}{\text{2}})-\left(d+h+\mu_{A}+\delta \right)({I_{A}}^{n}+\frac{N_{2}}{\text{2}})\right]dt+\sigma_{\text{3}}({I_{A}}^{n}+\frac{N_{2}}{\text{2}})\Delta B_{n},$

$O_{3}=\;h\left[\alpha \left(\text{1}-p_{\text{1}}\right){(E}^{n}+\frac{M_{2}}{\text{2}})+\delta \left(\text{1}-p_{\text{2}}\right)({I_{A}}^{n}+\frac{N_{2}}{\text{2}})-\left(d+h+\mu_{C}+\gamma \right)({I_{C}}^{n}+\frac{O_{2}}{\text{2}})\right]dt+\sigma_{\text{4}}({I_{C}}^{n}+\frac{O_{2}}{\text{2}})\Delta B_{n},$

$Q_{3}=\;h\left[\delta p_{\text{2}}({I_{A}}^{n}+\frac{N_{2}}{\text{2}})+\gamma ({I_{C}}^{n}+\frac{O_{2}}{\text{2}})-(d+h+\nu ){(R}^{n}+\frac{Q_{2}}{\text{2}})\right]dt+\sigma_{\text{5}}{(R}^{n}+\frac{Q_{2}}{\text{2}})\Delta B_{n},$

**Stage 4**

$\begin{array}{c} L_{4\;}=\;h\left[\;\Lambda -(d+h){(S}^{n}+L_{3\;})-\left(\beta_{A}{(S}^{n}+L_{3\;})({I_{A}}^{n}+N_{3\;})+\beta_{C}{(S}^{n}+L_{3\;})({I_{C}}^{n}+O_{3\;})\right)e^{-(d+h)\tau }+\nu {(R}^{n}+Q_{3\;})\right]dt+\sigma_{\text{1}}{(S}^{n}+L_{3\;})\Delta B_{n}, \end{array}$

$M_{4}=\;h\left[\;\left(\beta_{A}{(S}^{n}+L_{3\;})({I_{A}}^{n}+N_{3\;})+\beta_{C}{(S}^{n}+L_{3\;})({I_{C}}^{n}+O_{3\;})\right)e^{-(d+h)\tau }-(d+h+\alpha ){(E}^{n}+M_{3\;})\right]dt+\sigma_{\text{2}}{(E}^{n}+M_{3\;})\Delta B_{n},$

$N_{4}=h\left[\alpha p_{\text{1}}{(E}^{n}+M_{3\;})-\left(d+h+\mu_{A}+\delta \right)({I_{A}}^{n}+N_{3\;})\right]dt+\sigma_{\text{3}}({I_{A}}^{n}+N_{3\;})\Delta B_{n},$

$O_{4}=\;h\left[\alpha \left(\text{1}-p_{\text{1}}\right){(E}^{n}+M_{3\;})+\delta \left(\text{1}-p_{\text{2}}\right)({I_{A}}^{n}+N_{3\;})-\left(d+h+\mu_{C}+\gamma \right)({I_{C}}^{n}+O_{3\;})\right]dt+\sigma_{\text{4}}({I_{C}}^{n}+O_{3\;})\Delta B_{n},$

$Q_{4}=\;h\left[\delta p_{\text{2}}({I_{A}}^{n}+N_{3\;})+\gamma ({I_{C}}^{n}+O_{3\;})-(d+h+\nu ){(R}^{n}+Q_{3\;})\right]dt+\sigma_{\text{5}}{(R}^{n}+Q_{3\;})\Delta B_{n},$

Final stage


$$\begin{array}{c} \;\begin{array}{c} S^{n+\text{1}}=S^{n}+\frac{\text{1}}{\text{6}}[L_{1\;}+2L_{2\;}+2L_{3\;}+L_{4\;}]\\ E^{n+\text{1}}=E^{n}+\frac{\text{1}}{\text{6}}[M_{1\;}+2M_{2\;}+2M_{3\;}+M_{4\;}]\\ \begin{array}{c} {I_{A}}^{n+\text{1}}={I_{A}}^{n}+\frac{\text{1}}{\text{6}}[N_{1\;}+2N_{2\;}+2N_{3\;}+N_{4\;}]\\ \begin{array}{c} {I_{C}}^{n+\text{1}}={I_{C}}^{n}+\frac{\text{1}}{\text{6}}[O_{1\;}+2O_{2\;}+2O_{3\;}+O_{4\;}]\\ R^{n+\text{1}}=R^{n}+\frac{\text{1}}{\text{6}}[Q_{1\;}+2Q_{2\;}+2Q_{3\;}+Q_{4\;}] \end{array} \end{array} \end{array}\}\text{.} \end{array}$$ (25)

### 6.3. Stochastic non-standard computational method

The parametric perturbation model in equation (16) can be reformulated within a stochastic non-standard finite-difference (SNSFD) framework.


$$\begin{array}{c} d\text{S}(\text{t})=\left[\;\Lambda -(d+h)S(t)-\left(\beta_{A}SI_{A}+\beta_{C}SI_{C}\right)e^{-(d+h)\tau }+\nu R(t)\right]dt+\sigma_{\text{1}}SdB. \end{array}$$ (26)

$$\begin{array}{c} \frac{S^{n+\text{1}}-S^{n}}{h}=\left[\;\Lambda -(d+h)S^{n}(t)-\left(\beta_{A}S^{n}{I_{A}}^{n}+\beta_{C}S^{n}{I_{C}}^{n}\right)e^{-(d+h)\tau }+\nu R^{n}(t)\right]dt+\sigma_{\text{1}}S^{n}\Delta B_{n}\text{.} \end{array}$$ (27)

As in equation (27) for the stochastic NSFD process, we write equation (16) accordingly.


$$\begin{array}{c} S^{n+\text{1}}=\frac{S^{n}+h\nu +h\sigma_{\text{1}}S^{n}\Delta B_{n}}{\text{1}+h(d+h)S^{n}+h\left(\beta_{A}S^{n}{I_{A}}^{n}+\beta_{C}S^{n}{I_{C}}^{n}\right)e^{-(d+h)\tau }}\text{,} \end{array}$$ (28)

$$\begin{array}{c} E^{n+\text{1}}=\frac{E^{n}+h[\left(\beta_{A}S^{n}{I_{A}}^{n}+\beta_{C}S^{n}{I_{C}}^{n}\right)e^{-(d+h)\tau }]+h\sigma_{\text{2}}E^{n}\Delta B_{n}}{\text{1}+h(d+h+\alpha )E^{n}}\text{,} \end{array}$$ (29)

$$\begin{array}{c} {I_{A}}^{n+\text{1}}=\frac{{I_{A}}^{n}+h\alpha p_{\text{1}}E^{n}+\sigma_{\text{3}}h{I_{A}}^{n}\Delta B_{n}}{\text{1}+h(d+h+\mu_{A}+\delta )}\text{,} \end{array}$$ (30)

$$\begin{array}{c} {I_{C}}^{n+\text{1}}=\frac{{I_{C}}^{n}+h\alpha \left(\text{1}-p_{\text{1}}\right)E^{n}+h\delta \left(\text{1}-p_{\text{2}}\right){I_{A}}^{n}+h\sigma_{\text{4}}{I_{C}}^{n}\Delta B_{n}}{\text{1}+h(d+h+\mu_{C}+\gamma )}\text{,} \end{array}$$ (31)

$$\begin{array}{c} R^{n+\text{1}}=\frac{R^{n}+h\delta p_{\text{2}}{I_{A}}^{n}+h\gamma {I_{C}}^{n}+h\sigma_{\text{5}}R^{n}\Delta B_{n}}{\text{1}+h(d+h+\nu )}\text{.} \end{array}$$ (32)

where, n=0,1,2,… and $\Delta {\text{B}}_{\text{n}}=\Delta {\text{B}}_{t_{n+\text{1}}}-\Delta {\text{B}}_{t_{n}}$ is standardized distribution. i.e., $\Delta {\text{B}}_{\text{n}}\sim \text{N}(\text{0},\;\text{1}$).

Let $M=\frac{T}{\Delta \text{t}}$ denote the total number of time steps over the simulation interval [0,T], where Δt is the time-step size. The notation O(M) means that the total computational work increases linearly with the number of time steps. Since the SNSFD scheme performs a fixed number of calculations for the five state variables at each step, its computational complexity is O(M), similar to Euler-Maruyama and stochastic Euler methods. The stochastic Runge-Kutta method also has linear complexity but requires several intermediate stage evaluations at each step and is therefore computationally more expensive. The SNSFD scheme remains explicit and provides improved positivity and stability without a substantial additional computational burden.

Compared with the stochastic Euler, Euler–Maruyama, and stochastic Runge-Kutta methods, the SNSFD scheme is constructed to preserve the non-negativity, boundedness, and equilibrium properties of the continuous model. Its non-standard denominator function and nonlocal discretization reduce numerical instability and the occurrence of nonphysical negative solutions. Moreover, the method retains an explicit form and therefore requires relatively low computational effort.

### 6.4. Convergence analysis

The following theorems analyze the convergence properties of the scheme.

***Theorem 1***: For all n≥0 is positive invariant feasible region for eq. (28)–(32), the region $\Gamma =\{\left(S^{n}+E^{n}+{I_{A}}^{n}+{I_{C}}^{n}+R^{n}\right)\epsilon R_{+}^{\text{5}};\;S^{n}\geq \text{0},E^{n}\geq \text{0},{I_{A}}^{n}\geq \text{0},{I_{C}}^{n}\geq \text{0},R^{n}\geq \text{0};S^{n}+E^{n}+{I_{A}}^{n}+{I_{C}}^{n}+R^{n}\leq \frac{\Lambda }{d+h}\}$.

***Proof:***


$$\begin{array}{c} \frac{S^{n+\text{1}}-S^{n}}{\text{h}}=\left[\;\Lambda -(d+h)S^{n}(t)-\left(\beta_{A}S^{n}{I_{A}}^{n}+\beta_{C}S^{n}{I_{C}}^{n}\right)e^{-(d+h)\tau }+\nu R^{n}(t)\right]dt+\sigma_{\text{1}}S^{n}\Delta B_{n}. \end{array}$$

Similarly, for $E,\;I_{A},\;I_{C}\;and\;R$.


$$\begin{array}{c} \frac{E^{n+\text{1}}-E^{n}}{\text{h}}=\left[\;\left(\beta_{A}S^{n}{I_{A}}^{n}+\beta_{C}S^{n}{I_{C}}^{n}\right)e^{-(d+h)\tau }-(d+h+\alpha )E(t)\right]dt+\sigma_{\text{2}}E^{n}\Delta B_{n}, \end{array}$$

$$\begin{array}{c} \frac{{I_{A}}^{n+\text{1}}-{I_{A}}^{n}}{\text{h}}=\left[\alpha p_{\text{1}}E^{n}(t)-\left(d+h+\mu_{A}+\delta \right){I_{A}}^{n}\right]dt+\sigma_{\text{3}}{I_{A}}^{n}\Delta B_{n}, \end{array}$$

$$\begin{array}{c} \frac{{I_{C}}^{n+\text{1}}-{I_{C}}^{n}}{\text{h}}=\left[\alpha \left(\text{1}-p_{\text{1}}\right)E^{n}(t)+\delta \left(\text{1}-p_{\text{2}}\right){I_{A}}^{n}(t)-\left(d+h+\mu_{C}+\gamma \right){I_{C}}^{n}(t)\right]dt+\sigma_{\text{4}}{I_{C}}^{n}\Delta B_{n}, \end{array}$$

$$\begin{array}{c} \frac{R^{n+\text{1}}-R^{n}}{\text{h}}=\left[\delta p_{\text{2}}{I_{A}}^{n}+\gamma {I_{C}}^{n}-(d+h+\nu )R^{n}(t)\right]dt+\sigma_{\text{5}}R^{n}\Delta B_{n}. \end{array}$$

These equations are added up, then we get,


$$\begin{array}{c} \frac{{(S}^{n+\text{1}}+E^{n+\text{1}}+{I_{A}}^{n+\text{1}}+{I_{C}}^{n+\text{1}}+R^{n+\text{1}})-(S^{n}+E^{n}+{I_{A}}^{n}+{I_{C}}^{n}+R^{n})}{h}=\Lambda -(d+h)\left(S^{n}+E^{n}+{I_{A}}^{n}+{I_{C}}^{n}+R^{n}\right), \end{array}$$

$$\begin{array}{c} \frac{{(S}^{n+\text{1}}+E^{n+\text{1}}+{I_{A}}^{n+\text{1}}+{I_{C}}^{n+\text{1}}+R^{n+\text{1}})-(S^{n}+E^{n}+{I_{A}}^{n}+{I_{C}}^{n}+R^{n})}{h}\leq \Lambda -(d+h)\left(S^{n}+E^{n}+{I_{A}}^{n}+{I_{C}}^{n}+R^{n}\right), \end{array}$$

$$\begin{array}{c} {(S}^{n+\text{1}}+E^{n+\text{1}}+{I_{A}}^{n+\text{1}}+{I_{C}}^{n+\text{1}}+R^{n+\text{1}})\leq \frac{\Lambda }{\left(d+h\right)}. \end{array}$$

The obtained solution is bounded for all iterations n≥0.

***Theorem 2:*** Stability of the proposed numerical scheme is maintained as long as the eigenvalues are confined within the unit circle for all iteration steps n ≥ 0.

***Proof:*** let $A=\frac{S+h\nu R}{\text{1}+h(d+h)S+h\left(\beta_{A}SI_{A}+\beta_{C}SI_{C}\right)e^{-(d+h)\tau }},\;Y=\frac{E+h\left[\left(\beta_{A}SI_{A}+\beta_{C}SI_{A}\right)e^{-(d+h)\tau }\right]}{\text{1}+h(d+h+\alpha )E},$


$$\begin{array}{c} C=\frac{I_{A}+h\alpha p_{\text{1}}E}{\text{1}+h(d+h+\mu_{A}+\delta )},\;D=\frac{I_{C}+h\alpha \left(\text{1}-p_{\text{1}}\right)E+h\delta \left(\text{1}-p_{\text{2}}\right)I_{A}}{\text{1}+h(d+h+\mu_{C}+\gamma )},\;F=\frac{R+h\delta p_{\text{2}}I_{A}+h\gamma I_{C}}{\text{1}+h(d+h+\nu )}. \end{array}$$

The convergence of system (28)-(32) to its equilibrium is determined by the spectral radius ρ(J) of its Jacobian. The equilibrium is stable if ρ(J)<1, unstable if ρ(J)>1, and neutrally stable if ρ(J)=1.


$$\begin{array}{c} \frac{\partial A}{\partial S}=(\frac{\left(S+h\nu R)\left[h(d+h)+h\left(\beta_{A}I_{A}+\beta_{C}I_{C}\right)e^{-(d+h)\tau }\right]-(\text{1}+h(d+h)S+h\left(\beta_{A}SI_{A}+\beta_{C}SI_{C}\right)e^{-(d+h)\tau }\right)}{{\left(\text{1}+h(d+h)S+h\left(\beta_{A}SI_{A}+\beta_{C}SI_{C}\right)e^{-(d+h)\tau }\right)}^{\text{2}}}),\frac{\partial A}{\partial E}=\text{0}\;, \end{array}$$

$$\begin{array}{c} \frac{\partial A}{\partial I_{A}}=(\frac{(S+h\nu R)\left[h\left(\beta_{A}S\right)e^{-(d+h)\tau }\right]}{{\left(\text{1}+h(d+h)S+h\left(\beta_{A}SI_{A}+\beta_{C}SI_{C}\right)e^{-(d+h)\tau }\right)}^{\text{2}}}, \end{array}$$

$$\begin{array}{c} \frac{\partial A}{\partial I_{C}}=\left(\frac{(S+h\nu R)\left[h\left(\beta_{C}S\right)e^{-(d+h)\tau }\right]}{{\left(\text{1}+h(d+h)S+h\left(\beta_{A}SI_{A}+\beta_{C}SI_{C}\right)e^{-(d+h)\tau }\right)}^{\text{2}}}\right), \end{array}$$

$$\begin{array}{c} \;\frac{\partial A}{\partial R}=\left(\frac{h\nu }{\text{1}+h(d+h)S+h\left(\beta_{A}SI_{A}+\beta_{C}SI_{C}\right)e^{-(d+h)\tau }}\right), \end{array}$$

$$\begin{array}{c} \frac{\partial Y}{\partial S}=\frac{h\left(\beta_{A}I_{A}+\beta_{C}I_{C}\right)e^{-(d+h)\tau }}{\text{1}+h(d+h+\alpha )E}, \end{array}$$

$$\begin{array}{c} \frac{\partial Y}{\partial E}=\frac{\left(E+h\left[\left(\beta_{A}SI_{A}+\beta_{C}SI_{A}\right)e^{-(d+h)\tau }\right)h(d+h+\alpha )-(\text{1}+h(d+h+\alpha )E\right)}{[{\text{1}+h(d+h+\alpha )E]}^{\text{2}}}, \end{array}$$

$$\begin{array}{c} \frac{\partial Y}{\partial I_{A}}=\frac{h\left(\beta_{A}S\right)e^{-(d+h)\tau }}{\text{1}+h(d+h+\alpha )E}, \end{array}$$

$$\begin{array}{c} \frac{\partial Y}{\partial I_{C}}=\frac{h\left(\beta_{C}S\right)e^{-(d+h)\tau }}{\text{1}+h(d+h+\alpha )E}, \end{array}$$

$$\begin{array}{c} \frac{\partial Y}{\partial R}=\text{0},\;\frac{\partial C}{\partial S}=\text{0}, \end{array}$$

$$\begin{array}{c} \frac{\partial C}{\partial E}=\frac{h\alpha p_{\text{1}}}{\text{1}+h(d+h+\mu_{A}+\delta )}, \end{array}$$

$$\begin{array}{c} \frac{\partial C}{\partial I_{A}}=\frac{\text{1}}{\text{1}+h(d+h+\mu_{A}+\delta )}, \end{array}$$

$$\begin{array}{c} \frac{\partial C}{\partial I_{C}}=\text{0},\frac{\partial C}{\partial R}=\text{0}\frac{\partial D}{\partial S}=\text{0}, \end{array}$$

$$\begin{array}{c} \frac{\partial D}{\partial E}=\frac{h\alpha \left(\text{1}-p_{\text{1}}\right)}{\text{1}+h(d+h+\mu_{C}+\gamma )}, \end{array}$$

$$\begin{array}{c} \frac{\partial D}{\partial I_{A}}=\frac{h\delta \left(\text{1}-p_{\text{2}}\right)}{\text{1}+h(d+h+\mu_{C}+\gamma )}, \end{array}$$

$$\begin{array}{c} \frac{\partial D}{\partial I_{C}}=\frac{\text{1}}{\text{1}+h(d+h+\mu_{C}+\gamma )}, \end{array}$$

$$\begin{array}{c} \frac{\partial D}{\partial R}=\text{0},\frac{\partial F}{\partial S}=\text{0},\frac{\partial F}{\partial E}=\text{0}, \end{array}$$

$$\begin{array}{c} \frac{\partial F}{\partial I_{A}}=\frac{h\delta p_{\text{2}}}{\text{1}+h(d+h+\nu )}, \end{array}$$

$$\begin{array}{c} \frac{\partial F}{\partial I_{C}}=\frac{h\gamma }{\text{1}+h(d+h+\nu )}, \end{array}$$

$$\begin{array}{c} \frac{\partial F}{\partial R}=\frac{\text{1}}{\text{1}+h(d+h+\nu )}. \end{array}$$

At disease free equilibrium point $E_{\text{1}}=(\frac{\Lambda }{d+h},\text{0},\text{0},\text{0},\text{0})$. The Jacobean matrix is


$$\begin{array}{c} J\left(E_{\text{1}}\right)=[\begin{array}{ccccc} \frac{\left(\frac{\Lambda }{d+h}\right)\left[h(d+h)\right]-(\text{1}+h\Lambda )}{(\text{1}+h\Lambda {)}^{\text{2}}} & \text{0} & \frac{\left(\frac{\Lambda }{d+h}\right)\left[h\left(\beta_{A}\frac{\Lambda }{d+h}\right)e^{-(d+h)\tau }\right]}{(\text{1}+h\Lambda {)}^{\text{2}}} & \frac{\left(\frac{\Lambda }{d+h}\right)\left[h\left(\beta_{C}\frac{\Lambda }{d+h}\right)e^{-(d+h)\tau }\right]}{(\text{1}+h\Lambda {)}^{\text{2}}} & \frac{h\nu }{\text{1}+h(d+h)\frac{\Lambda }{d+h}}\\ \text{0} & \text{0} & \text{0} & \text{0} & \text{0}\\ \text{0} & \frac{h\alpha p_{\text{1}}}{\text{1}+h(d+h+\mu_{A}+\delta )} & \frac{\text{1}}{\text{1}+h(d+h+\mu_{A}+\delta )} & \text{0} & \text{0}\\ \text{0} & \frac{h\alpha \left(\text{1}-p_{\text{1}}\right)}{\text{1}+h(d+h+\mu_{C}+\gamma )} & \frac{h\delta \left(\text{1}-p_{\text{2}}\right)}{\text{1}+h(d+h+\mu_{C}+\gamma )} & \frac{\text{1}}{\text{1}+h(d+h+\mu_{C}+\gamma )} & \text{0}\\ \text{0} & \text{0} & \frac{h\delta p_{\text{2}}}{\text{1}+h(d+h+\nu )} & \frac{h\gamma }{\text{1}+h(d+h+\nu )} & \frac{\text{1}}{\text{1}+h(d+h+\nu )} \end{array}]. \end{array}$$

The eigenvalues are $\lambda_{\text{1}}=\frac{\left(\frac{\Lambda }{d+h}\right)\left[h(d+h)\right]-(\text{1}+h\Lambda )}{(\text{1}+h\Lambda {)}^{\text{2}}}<\text{1}$, $\lambda_{\text{2}}=\text{0}<\text{1},$ $\lambda_{\text{3}}=\frac{\text{1}}{\text{1}+h(d+h+\mu_{A}+\delta )}<\text{1}$,

$\lambda_{\text{4}}=\frac{\text{1}}{\text{1}+h(d+h+\mu_{C}+\gamma )}$, $\lambda_{\text{5}}=\frac{\text{1}}{\text{1}+h(d+h+\nu )}$.

At disease existing point, we see below:


$$\begin{array}{c} J\left(E_{\text{2}}\right)=[\begin{array}{ccc} \frac{\text{1}}{\text{1}+hcI^{*}\left(\text{1}+\gamma I^{*}\right)e^{-\mu \tau }+k\mu } & \frac{\left(S^{*}+ha+h\alpha R^{*}\right)\left(hc+\text{2}hc\gamma I^{*}e^{-\mu \tau }\right)}{{\left(\text{1}+hcI^{*}\left(\text{1}+\gamma I^{*}\right)e^{-\mu \tau }\right)}^{\text{2}}} & \frac{h\alpha }{\text{1}+hcI^{*}\left(\text{1}+\gamma I^{*}\right)}\\ \frac{hcI^{*}\left(\text{1}+\gamma I^{*}\right)e^{-\mu \tau }}{\text{1}+h(\beta +\mu +\delta -b)} & \frac{\text{1}+hcS^{*}+\text{2}hcS^{*}\gamma I^{*}+hb}{\text{1}+h(\beta +\mu +\delta -b)} & \text{0}\\ \text{0} & \frac{h\beta }{\text{1}+h(\alpha +\beta )} & \frac{\text{1}}{\text{1}+h(\alpha +\beta )} \end{array}]. \end{array}$$

By using MATLAB for plotting the largest eigenvalues of endemic equilibrium points and values of parameters presented in [20].

## 7. Results

**Fig 2:**
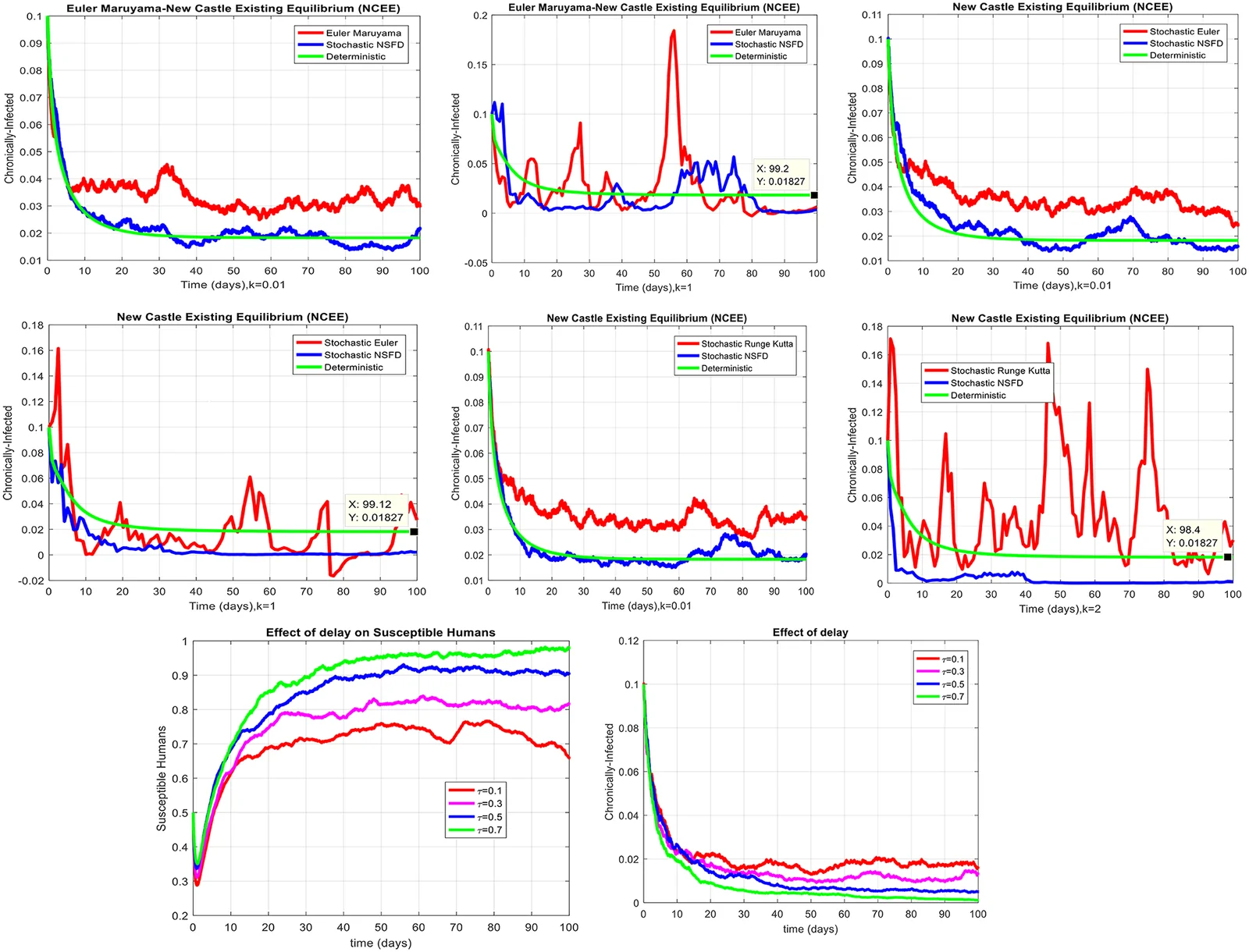
In this chronically infected group, when using all three methods, Euler–Maruyama, Euler, and Runge-Kutta with NSFD, for small step size (k=0.01), all these methods result in stable convergence, while for larger step sizes, such as k=1 or k=2, divergence is observed. Furthermore, the dynamics of both susceptible and chronically infected populations are strongly affected by the time delay parameter.

This section examines the fundamental computational properties of the non-linear model given by (1)-(5). The goal is to use numerical methods to describe essential dynamical properties of the Newcastle disease virus model: positivity, boundedness, and dynamic consistency at its steady states. Further confirmation of stability in the discretized system is addressed by an examination of the magnitude of its eigenvalues. Graphical results depicting the equilibrium points are presented. The comparison graphs show that all methods provide similar approximations for sufficiently small time steps. However, conventional methods may yield unstable or negative solutions as the step size increases, whereas the SNSFD scheme more effectively preserves non-negativity and boundedness, and ensures stable convergence. Comparison plots showing small variations in time-step for the Stochastic Euler, Stochastic Runge-Kutta, and SNSFD schemes are presented. The numerical simulations show that time delays, τ, significantly affect both the susceptible and infected compartments. For a time delay of τ=0.7, the susceptible population exhibits a positive, increasing trend. By contrast, the chronically infected population under this delay generates a negative, decreasing trend which converges to zero. Indeed, Figs 2(a), 2(c), and 2(e) show that all three numerical methods converge to the predicted equilibrium points for sufficiently small time steps (in days). On the other hand, unstable, unbounded and nonphysical (negative) solutions are obtained for slightly larger time steps, as illustrated in Figs 2(b), 2(d) and 2(f). Finally, the effects of variation in the delay parameter on the susceptible and chronically infected populations are displayed graphically in Figs 2(g) and 2(h), respectively.

The reproduction number $R_{\text{0}}$ determines whether Newcastle disease can invade the population. If $R_{\text{0}}<\text{1}$, the infection is expected to disappear, whereas $R_{\text{0}}>\text{1}$ indicates possible persistence. Unlike the classical SEIR model, the present formulation includes acute and chronic infection, delay, and stochastic effects. Direct validation is limited by the lack of suitable white-winged parakeet data.

### 7.1. Practical implementation with epidemiological data

For practical implementation, surveillance data on infected birds, recoveries, disease-related deaths, and population size can be used to estimate the model parameters through least-squares or likelihood-based methods. The estimated model can then be validated by comparing simulated and observed disease trends. Because suitable longitudinal data for white-winged parakeets were unavailable, the present study focuses on theoretical and numerical analyses; future work will consider data-driven calibration and validation.

## 8. Conclusion

This research investigates a stochastic delayed mathematical model to simulate the dynamics of infectious disease spread, with potential applications to understanding the propagation of the Newcastle virus. The basic system is first formulated as a set of stochastic delay differential equations. An extended version of this model, incorporating parametric perturbations to represent diverse states and transition probabilities, is presented here in full. Numerically, the authors used the standard methods of Stochastic Euler, Stochastic Runge-Kutta, and Euler-Maruyama; however, these methods have proved unsatisfactory for reliably replicating the model’s dynamic properties. To overcome the drawback of this limitation, a new computational scheme was proposed. This approach, being a stochastic delayed analogue of NSFD techniques, successfully maintained the model’s biologically feasible region without needing restrictive conditions on parameters. The numerical simulations show that time delays indeed significantly affect both the susceptible and infected population compartments: an increase in delay is associated with an increase in the susceptible population, corresponding to a decrease in infectivity that may reach zero, and vice versa.
